# Elucidating the Role of Noncovalent Interactions in Favipiravir, a Drug Active against Various Human RNA Viruses; a ^1^H-^14^N NQDR/Periodic DFT/QTAIM/RDS/3D Hirshfeld Surfaces Combined Study

**DOI:** 10.3390/molecules28083308

**Published:** 2023-04-07

**Authors:** Jolanta Natalia Latosińska, Magdalena Latosińska, Janez Seliger, Veselko Žagar, Tomaž Apih, Paweł Grieb

**Affiliations:** 1 Faculty of Physics, Adam Mickiewicz University, Uniwersytetu Poznańskiego 2, 61-614 Poznań, Poland; 2 Faculty of Mathematics and Physics, University of Ljubljana, Jadranska 19, 1000 Ljubljana, Slovenia; 3 “Jožef Stefan” Institute, Jamova 39, 1000 Ljubljana, Slovenia; 4 Department of Experimental Pharmacology, Mossakowski Medical Research Institute, Polish Academy of Science, Adolfa Pawińskiego 5, 02-106 Warszawa, Poland

**Keywords:** favipiravir, interactions pattern, hydrogen bonds, binding modes of FVP, COVID-19, Ebola, SARS-CoV-2, structure

## Abstract

Favipiravir (6-fluoro-3-hydroxypyrazine-2-carboxamide, FPV), an active pharmaceutical component of the drug discovered and registered in March 2014 in Japan under the name Avigan, with an indication for pandemic influenza, has been studied. The study of this compound was prompted by the idea that effective processes of recognition and binding of FPV to the nucleic acid are affected predominantly by the propensity to form intra- and intermolecular interactions. Three nuclear quadrupole resonance experimental techniques, namely ^1^H-^14^N cross-relaxation, multiple frequency sweeps, and two-frequency irradiation, followed by solid-state computational modelling (density functional theory supplemented by the quantum theory of atoms in molecules, 3D Hirshfeld Surfaces, and reduced density gradient) approaches were applied. The complete NQR spectrum consisting of nine lines indicating the presence of three chemically inequivalent nitrogen sites in the FPV molecule was detected, and the assignment of lines to particular sites was performed. The description of the nearest vicinity of all three nitrogen atoms was used to characterize the nature of the intermolecular interactions from the perspective of the local single atoms and to draw some conclusions on the nature of the interactions required for effective recognition and binding. The propensity to form the electrostatic N−H···O, N−H···N, and C−H···O intermolecular hydrogen bonds competitive with two intramolecular hydrogen bonds, strong O−H···O and very weak N−H···N, closing the 5-member ring and stiffening the structure, as well as π···π and F···F dispersive interactions, were analysed in detail. The hypothesis regarding the similarity of the interaction pattern in the solid and the RNA template was verified. It was discovered that the -NH_2_ group in the crystal participates in intermolecular hydrogen bonds N–H···N and N–H···O, in the precatalytic state only in N–H···O, while in the active state in N–H···N and N–H···O hydrogen bonds, which is of importance to link FVP to the RNA template. Our study elucidates the binding modes of FVP (in crystal, precatalytic, and active forms) in detail and should guide the design of more potent analogues targeting SARS-CoV-2. Strong direct binding of FVP-RTP to both the active site and cofactor discovered by us suggests a possible alternative, allosteric mechanism of FVP action, which may explain the scattering of the results of clinical trials or the synergistic effect observed in combined treatment against SARS-CoV-2.

## 1. Introduction

The coronavirus disease-2019 (COVID-19), an infectious disease associated with novel severe acute respiratory syndrome coronavirus-2 (SARS-CoV-2) [1,2], originated in December 2019 in the Hubei Province of China. Since the spread of the COVID-19 pandemic throughout the world, efforts to find effective treatments have been persistently undertaken. The recent endeavours in the field of drug design are aimed at discovering old or newly designed drugs which could be effective for the treatment of this contagious disease caused by different SARS-CoV-2 variants (Alpha, Beta, Gamma, Delta, Omicron) and subvariants, especially so-called variants of concern (VOC) lineages under WHO monitoring (BA.5, BA.2.75, BA.4.6, XBB, BA.2.3.20) [3].

**Figure 1:**
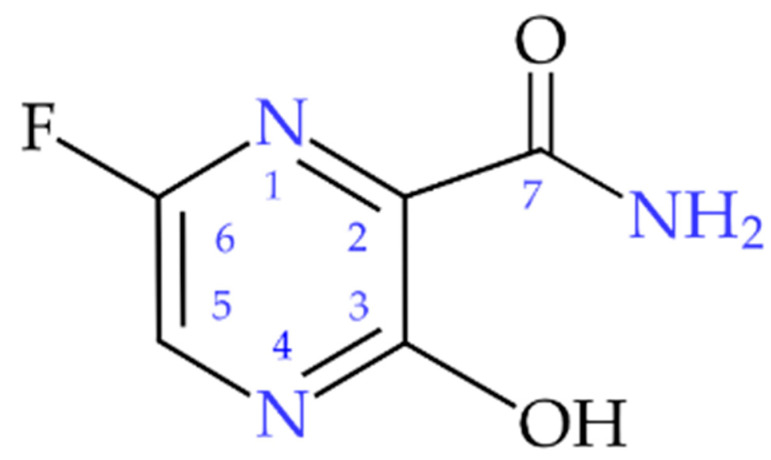
The structural formula of Favipiravir (6-fluoro-3-hydroxypyrazine-2-carboxamide, FVP).

Favipiravir (6-fluoro-3-hydroxypyrazine-2-carboxamide, FPV, T-705), Figure 1, is an active pharmaceutical ingredient of the drug discovered by the Japanese company Toyama Kagaku Kōgyō and registered in March 2014 in Japan under the name Avigan, with an indication for pandemic influenza [4,5,6,7].

It is well known for its in vitro activity towards OTV-resistant influenza A, B, and C viruses as well as flavi-, alpha-, filo-, bunya-, arena-, and noroviruses [8,9]. Preclinical trials performed before and after its first registration have shown strong FPV activity against several viruses containing negative-strand RNA as genetic material and causing serious human diseases [10], such as Ebola [11], Lassa virus [12], West Nile Fever [13], Zika [14], tick-borne encephalitis [15], and even rabies [16]. The antiviral activity of FPV consists of disrupting the normal function of the RNA-dependent RNA polymerase (RdRP), which produces a “mirror image” of a viral RNA, later used as a positive-sense RNA needed to synthesize multiple copies of nascent virus RNAs. RdRP malfunction in the presence of FPV depends on the intracellular phosphorylation of the drug to its active form (FPV triphosphate), a false nucleoside that is built by the viral RdRP into the nascent viral RNA, resulting in a “defective”, mutated RNA. Thus, FVP is, in fact, a prodrug. It has only recently been discovered that the dominant mechanism of action of FVP is unlikely to be related to the delayed chain termination but to its action as a strong viral mutagen not inducing mutations beneficial for the virus [17], likely because these mutations cannot be effectively repaired by the viral proteins performing a “technical check” on the replication of virus’ genetic material. Importantly, FVP is not used by human RNA polymerases, making it a low-toxicity drug. The therapeutic effect of FPV is a result of the accumulation of mutations in the replicated RNA of nascent viruses which lose their ability to proliferate. In Africa, FPV (along with the US-made drug Remdesivir, RSV, brand name Veklury) was clinically tested a few years ago as a potential drug for Ebola. Initially, results were promising, but the Ebola epidemic subsided and the trials were discontinued due to the lack of patients. However, in 2022, about 60 new cases of Ebola virus disease (EVD) were discovered in Mubende, Kassanda, and Kampala Districts in the Central Region, and Bunyangabu, Kagadi, and Kyegegwa Districts in the Western Region of Uganda.

After the identification of the new virus, SARS-CoV-2, FVP was one of the first drugs whose effectiveness and safety were tested on the COVID-19 disease caused by this virus. This drug, along with the aforementioned RSV, raised the greatest hopes for the effective treatment of COVID-19. Compared to Molnupiravir or Nirmatrelvir, FVP is less potent against the SARS-CoV-2 virus in vitro [18,19] but similarly potent in animal models of SARS-CoV-2 [20,21]. Currently, FPV is registered as a drug for COVID-19 in China, India, and Russia, and it is in the process of being registered in Japan and several other countries. It has been also approved for the treatment of COVID-19 infections in Turkey, the KSA, Serbia, Hungary, and Egypt [22].

According to the ClinicalTrials.gov database, there are currently 58 completed, ongoing, or planned clinical trials in many countries around the world, assessing the efficacy and safety of FPV in COVID-19; these include trials conducted in Europe (including Italy, Denmark, and Hungary), USA, Canada, and Australia. FPV is now approved by some countries, including India, for COVID-19 treatment [23]. FPV therapy with safe oral doses is effective for the treatment of outpatients with mild to moderate COVID-19 infection. Moreover, results regarding viral load reduction and an improvement in the radiological and clinical outcomes in COVID-19 patients are remarkable [24,25]. FPV is a very promising drug due to its mechanism of action, preclinical results, high degree of safety in humans, bioavailability, good treatment progress, and manufacturing reliability.

Despite such promising applications, the FVP compound has been poorly studied and only scarce physicochemical data on this compound are available. The limiting factor was the FPV patent protection, which has fortunately now expired. The drug has a formal status of a generic, thanks to which it can be offered not only by the original manufacturer (Fujifilm Holdings, Japan) but also by alternative manufacturers (e.g., Avifavir, Coronavir, and Areplivir (RU); Avigan (JP); FabiFlu, Favijaj, Fluguard, Ciplenza, and Covihalt (IN); Favira (BD); Avipiravir (EG); and Reeqonus (CA)). This study focuses on the assessment of the binding capacity of FVP molecules and their impact on biological activity. The pyrazine heterocyclic ring and three functional groups (fluoro, hydroxide, and amide) in the FVP molecule allow it to engage in weak van der Waals, steric, or stacking interactions while the presence of the three donor (amide and hydroxide) or five acceptor atoms (i.e., second-order elements: two-aromatic-ring nitrogen, the oxygen of amide, hydroxide, and fluorine) in one molecule facilitates the formation of hydrogen bonds. According to the Etter rule [26,27], the molecule tends to form as many hydrogen bonds as possible. FVP can theoretically realize even as many as fifteen different types of hydrogen bonds (both intra- and intermolecular). Thus, it should easily bind to protein, which is pharmaceutically very attractive. However, the existence of the intramolecular OH···O bond limits the number of donors (protons) available, and, thus, only four strong hydrogen bonds (through two donor hydrogen atoms of the amide group, and any two of the five remaining acceptor atoms two N, two O, and one F) seem feasible. The fundamental question is what kinds of bonds are indeed formed in the crystal structure, what their strength is, which of them involve the acceptor atoms (especially nitrogen), and to what extent their individual bonding abilities facilitate the formation of the protein–ligand complex. The interplay of long-range (electrostatic: pairwise additive and repulsive/attractive, inductive: non-additive and attractive, and dispersive: non-additive and attractive) and short-range (exchange and repulsion: non-additive and opposite sign) interactions is not only crucial for efficient protein–ligand binding but also changes the translation patterns.

The non-destructive spectroscopy that delivers a unique and complementary X-ray description of the nearest vicinity of atoms is ^1^H-^14^N nuclear quadrupole double resonance. Being more sensitive to the changes in the local environment than NMR, IR or powder X-ray diffraction (PXRD) [28,29,30], ^1^H-^14^N NQDR is a particularly valuable tool which delivers high-quality local information and provides a validation criterion for the correct reproduction of the total electron density distribution obtained by quantum chemistry calculations. Moreover, ^1^H-^14^N NQDR combined with modern approaches including density functional theory (DFT), Bader’s quantum theory of atoms in molecules (QTAIM) [31], Johnson’s reduced density gradient (RDS) [32], Spackman’s Hirshfeld surface-based, [33,34], electrostatic potential (ESP) [35] provide a deeper insight into the three-dimensional crystalline packing at the atomic and molecular levels [36,37,38]. An in-depth study of the interaction pattern should help predict the ability of FVP to bind to specific biological targets (proteins and nucleic acids: DNA, RNA) and thus assess its pharmaceutical attractiveness. The possible competition between interactions raises the question of whether the experimentally observed local hydrogen-bonded environment in the solid state corresponds to the most likely interactions required for effective processes of FPV recognition and binding to the proteins or nucleic acids (DNA or RNA). We anticipated that our study would clarify these aspects and help in outlining the directions of synthesis of new active pharmaceutical ingredients that would be promising in the fields of the chemical industry and drug design of more effective analogues that are promising for COVID-19 and Ebola treatments.

## 2. Results and Discussion

### 2.1. 1H-^14^N NQDR Spectrum

The ^14^N NQR frequencies in FVP lie in the low-frequency range below 4 MHz, and direct NQR measurement on a small sample (approx. 0.5g) is not possible due to a very low signal-to-noise ratio. Instead, the ^14^N NQR frequencies were determined indirectly by measuring the proton NMR signal using nuclear quadrupole double resonance techniques. A multistep procedure was used.

*In the first step*, the ^1^H-^14^N cross-relaxation (CR)[39] spectrum was measured using fast field cycling (FFC) relaxometry. In a standard CR experiment, three successive time intervals with different values of a static magnetic field are used:

(i) Polarization field $B_{P}$ for time $t_{P}.$ Typically,$\;t_{P}\geq 3T_{1H}\left(B_{P}\right)$, where $T_{1H}\left(B_{P}\right)$ is the proton spin-lattice relaxation time in the field $B_{P}$ so that an equilibrium proton magnetization is established.
(ii) Relaxation (also called “mixing”) field $B_{R}$ for time τ. Here the proton magnetization relaxes towards the equilibrium value with a time constant $T_{1H}\left(B_{R}\right)$.
(iii) Acquisition field $B_{A}$, at which the proton NMR signal amplitude, which is proportional to the proton magnetization at the end of $B_{R}$ interval, is measured.

By repeating the experiment with a different relaxation field $B_{R}$ and relaxation time τ, the full dispersion of spin-lattice relaxation time $T_{1H}\left(B_{R}\right)$ is determined. At values of magnetic field $B_{R}$, where the proton NMR Larmor frequency $\nu_{H}$ coincides with one of the ^14^N NQR frequencies $\;\nu_{Q}$ or $\nu_{H}=\frac{\gamma_{H}}{2\pi }B_{R}=\nu_{Q}$, a decrease in $T_{1H}\left(B_{R}\right)$ is often observed. This phenomenon is well known as “quadrupole dips” [40] and is often used to indirectly determine the NQR frequencies.

In the case of FVP, the initial measurements have shown that $T_{1H}\left(B_{P}\right)$ is longer than 100 s in the polarization field $B_{P}=0.5\;T$, which is typically used in our laboratory. At such long values, the power consumption of the relaxometer is too high, and the sample cannot be properly polarised. Additionally, long $T_{1H}$ makes measurement very time-consuming.

Therefore, we modified the technique in such a way that the polarization period was removed from the experiment ($B_{P}=0$), and the proton magnetization was zero at the beginning of the $B_{R}$ interval. Then, the proton magnetization grows towards the equilibrium value as $M=M_{0}\left(1-e^{-\frac{\tau }{T_{1H}\left(B_{R}\right)}}\right)$. In addition, for values of $B_{R}$ at which the ^1^H proton NMR transition frequency $\nu_{H}\;$matches one of the ^14^N NQR transition frequencies $\nu_{Q}$, the proton system is additionally polarised by the transfer of polarization from nitrogen NQR levels to proton NMR levels with a characteristic cross-relaxation time $T_{CR}$. If $T_{CR}<T_{1H}\left(B_{R}\right)$, additional “quadrupole peaks” thus arise in the CR spectrum.

**Figure 2:**
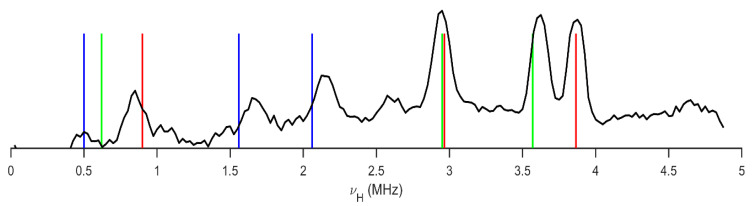
^1^H-^14^N cross-relaxation spectrum in favipiravir, as measured by the modified cross-relaxation technique at T = 295 K. The quadrupole peaks are Zeeman-shifted and powder-broadened with respect to true ^14^N NQR frequencies, determined using a technique with multiple frequency sweeps and two-frequency irradiation (shown as horizontal lines).

The ^1^H-^14^N cross-relaxation spectrum of FVP, as measured by the modified cross-relaxation technique, is presented in Figure 2. Here, a fixed value of τ = 3 s was used, and the measurement was repeated so that the “relaxation” of the magnetic field $B_{R}$ was scanned in steps of 0.585 mT, which corresponds to a step in the proton NMR frequency $\Delta \nu_{H}$ equal to 25 kHz. The proton NMR frequency in the acquisition field $B_{A}$ was 9.25 MHz.

Three strong peaks centred at the frequencies of 3.870 MHz, 3.610 MHz, and 2.940 MHz are clearly observed. These peaks are observed in the frequency range in which the higher ^14^N NQR frequencies ν_+_ and ν_−_ of pyrazine nitrogen are usually observed. Since there are two non-equivalent pyrazine nitrogen atoms in the molecule, we expect to observe four peaks. It is possible that two peaks overlap within the experimental resolution.

Two somewhat weaker peaks are observed at the frequencies of 2.140 MHz and 1.610 MHz. In this frequency range, the amide nitrogen NQR frequencies ν_+_ and ν_−_ are usually observed. Below 1 MHz, there is a well-observed peak at 830 kHz, but the signal-to-noise ratio at low $\nu_{H}$ is too low to observe two additional peaks, which are expected close to 500 kHz. Although the peaks are well located in the spectrum, the resolution is relatively low due to the Zeeman-powder broadening of the quadrupole peaks. Additionally, the CR peaks are Zeeman-shifted with respect to the pure NQR frequencies in the zero field [41].

*In the second step* we, therefore, used the techniques of multiple frequency sweeps and two-frequency irradiation [42,43] for a more precise determination of the frequencies of the NQR triplets ν_+_, ν_−_, and ν_0_ at the three nitrogen positions in an FVP molecule. In the search for the ^14^N NQR frequencies at the pyrazine nitrogen positions, we applied multiple frequency sweeps of the *rf* magnetic field in the frequency range 2.8–4 MHz and performed the ν_H_ scan. We clearly observed two dips in the ν_L_ dependence of the magnitude of the proton NMR signal at the end of the magnetic field cycle at the frequencies of 620 kHz and 900 kHz. These are the lowest frequencies ν_0_ at the two pyrazine nitrogen positions. Then, we fixed the low magnetic field within the dip at ν_H_ = 620 kHz and selectively varied the upper and the lower-frequency limit. When the upper-frequency limit passes 3570 kHz from above, the magnitude of the proton NMR signal increases. Similarly, when the lower-frequency limit passes 2.950 MHz from below, the proton NMR signal increases. Therefore, the frequencies of 3.570 MHz, 2.950 MHz, and 620 kHz are within the experimental resolution of this technique, i.e., the ^14^N NQR frequencies at the one pyrazine nitrogen position. We tried to refine these values with the application of the two-frequency irradiation technique; however, within the experimental resolution of this technique, ±5 kHz, we obtained the same results. Similarly, we determined the ^14^N NQR frequencies at the second pyrazine position and the amide nitrogen position. The obtained results are presented in Table 1.

The experimental ^1^H-^14^N NQDR spectrum of FVP consists of nine signals, Figure 2, which must be grouped into three groups for each of the three ^14^N sites, as shown in Table 1. The NQR parameters e^2^Qqh^−1^ and η, calculated from these data using Equations (2) and (3), are listed in Table 1. The number of resonance lines indicates that the molecules in the FPV crystal unit cell are equivalent and symmetry-related. Moreover, the NQR spectrum of FVP is normally broadened, with no evidence of structural disorder.

The electronic environment of all *three* nitrogen nuclei in the FVP molecule is different, so they are *chemically* non-equivalent. All nitrogen sites have distinctly different e^2^Qqh^−1^ but very close η; the latter is rather unusual. The low ν_+_ and e^2^Qqh^−1^ as well as the characteristic resonant frequencies observed in the spectrum are typical of amide nitrogen (–NH_2_). The ν_+_ frequency of –NH_2_ is substantially lower than that of aniline (3.244 MHz) and close to those in acetamide (2.105 MHz) or formamide (1.920 MHz) [45], which suggests a strong conjugation between the amide nitrogen and the adjacent carbon in FVP. Additionally, the value of η calculated from the ^14^N NQR spectrum, closer to that of –N= than the –NH_2_ type of nitrogen, suggests that the electron density at –NH_2_ is significantly shifted due to the involvement of this moiety in the intermolecular interactions. Both NQR parameters in FVP are not only close to those observed for acetamide or formamide having hydrogen bonds N–H···O but also closer to that of cytosine [46] having two kinds of hydrogen bonds N–H···O and N–H···N [30], than to that of aminopyridines with only weaker N–H···N bonds. It points to the participation of –NH_2_ in FVP in at least two kinds of hydrogen bonds, most likely N–H···O and N–H···N. Thus, a basic analysis of the NQR spectrum suggests that the set of resonant lines resulting in the lowest e^2^Qqh^−1^ can be assigned to the amide nitrogen –NH_2_, while the sets resulting in the two higher ones can be assigned to the pyrazine nitrogen atoms –N(1)= and –N(4)=. An unambiguous assignment of pyrazine nitrogen signals based solely on the NQR spectra is not possible.

**Figure 3:**
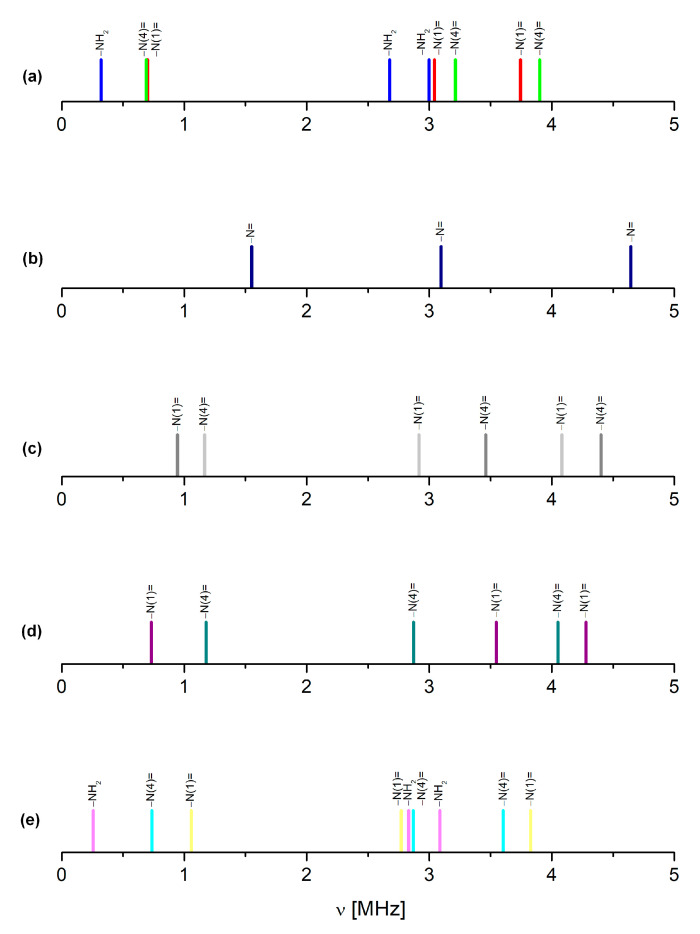
A comparison of the frequency shifts in the ^1^H-^14^N NQDR spectrum caused by substituents in single molecules of (**a**) FVP, (**b**) pyridazine, (**c**) 2-fluoropyrazine, (**d**) 2-hydroxy-pyrazine, and (**e**) pyrazine-2-carboxamide.

The substituents effect, analysed directly using the NQR parameters for the structurally related compounds or modelled using the calculations for the single molecules, should be helpful in the assignment of the pyrazine signals. Due to the lack of experimental data (NQR spectra are available only for unsubstituted pyrazine [44]), the substituent effect could be only modelled. The results are summarized in Table 2 and shown in Figure 3.

The fluorine and hydroxyl groups substituted directly into the pyrazine ring at the C(6) and C(3) positions, respectively, are both σ electron-withdrawing and π-electron-donating. However, due to their symmetrical position in relation to the pyrazine nitrogen atoms, they have a competitive effect and act very similarly on the –N(1)= and –N(4)= nitrogen atoms. As a consequence, the shifts in the NQR frequencies and the values of parameters for both –N(1)= and –N(4)= differ only slightly, as shown in Table 2. The amide group substituted at the C(2) position of the ring also acts as an σ electron-withdrawing group, and its nitrogen is an excellent π-donor primarily to the carboxylic carbon. Therefore, it affects the electron density distribution in the ring but has a slightly stronger influence on –N(4)= in the meta position, than –N(1)= in the ortho position. Surprisingly, the amide group is quite an important factor in determining the position of the resonance lines in the spectrum, as shown in Figure 3. It means that the effect of the resultant substituent in the FVP molecule is complicated by a combination of nonadditive overlap and drift effects, resulting in a significant decrease in both NQR parameters (e^2^Qqh^−1^ and η) on both pyrazine nitrogen atoms. The single-molecule calculations, performed for the optimized and “solid” molecule (i.e., cut out of the crystal), reproduce this effect only roughly. The Pearson correlation coefficient is not very high, the scattering is quite large, and the NQR frequencies derived from these parameters are overestimated. This is a result of neglecting the further surroundings, in particular the nearest neighbouring molecules. The simulation of the ^14^N NQR spectra of the other heterocyclic compounds showed that NQR parameters were very sensitive, even to the influence of the distant proximity of nitrogen atoms [29,38]. This raises a suspicion that despite reasonable values of NQR parameters, the assignment of the signals based on single-molecule calculations in FVP may be questionable.

**Figure 4:**
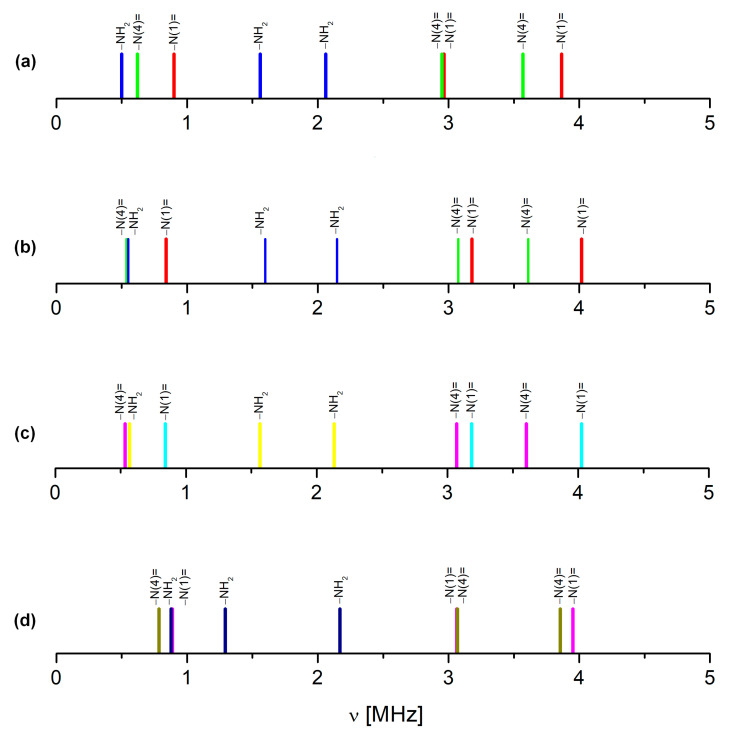
The ^1^H-^14^N NQDR spectrum of FVP (**a**) experimental and calculated assuming structures (**b**) CCDC 2048895, (**c**) CCDC 2106318, and (**d**) CCDC 969968.

The solid-state calculations that take into account all intermolecular interactions require a high-quality reliable crystal structure. The Cambridge structural database (CSD) contains four FVP crystalline structures for two different polymorphs (three polymorph I and one polymorph II; all enol-like forms). Polymorph I of FPV crystallizes in the orthorhombic space group *Pna*2_1_, with four equivalent molecules in the unit cell whose dimensions vary with temperature from a= 9.06680 Å, b = 14.85080 Å, and c = 4.57550 Å at 100 K (CCDC 2048895 [47]) or a = 9.14400 Å, b = 14.87000 Å, and c = 4.55730 Å at 100 K (CCDC 2106318 [48]), to a = 9.11060 Å, b = 14.76190 Å, and c = 4.69100 Å at 296 K (CCDC 969968 [49]). Polymorph II of FPV crystallizes in the tetragonal space group P42/n with four equivalent molecules in the unit cell of dimensions a = 9.28050 Å, b = 9.28050 Å, and c= 14.64910 Å at 298 K (CCDC 2047143 [50]). The ^14^N NQDR spectrum indicates the presence of only one polymorphic form in the sample; however, it is not possible to infer from it which one exactly. Therefore, all of these structures were used as inputs for the DFT/GGA calculations. The results of the calculations assuming the structures CCDC 2048895, 2106318, and 969968 as inputs, are significantly better than those obtained assuming CCDC 2047143 (the Pearson correlation is higher and the scattering is smaller), as shown in Table 3 and Table 4, and Figure 4. Thus, the simulated spectrum, much closer to the experimental one, clearly indicates the presence of polymorph I in the studied sample.

The differences between the experimental and calculated NQR parameters are rather small for the pyrazine nitrogen but quite large for the amide nitrogen, regardless of the level of theory and type of structure assumed. While e^2^Qq/h is quite well reproduced, the asymmetry parameter is significantly, almost twice, overestimated. This leads to the resonance frequencies ν_−_ and ν_0_ for –NH_2_ calculated from the NQR parameters and Equations (4)–(6) being far from the experimental values. The ν_+_ frequency for –N(4)= also deviates from the experimental value. There are two main reasons for this phenomenon: one is related to the quality of the crystallographic structure and associated with thermal effects, and the other is related to the nature of intermolecular interactions. X-ray crystallography at the usual resolution does not permit the exact assessment of the positions of light atoms, mainly because their contributions to diffraction are weak. In addition, the atoms oscillate around their equilibrium positions and the amplitude of the oscillations strongly depends on temperature (the lower the temperature, the smaller the oscillations).

At higher temperatures, the electron density is spread out over a larger volume, resulting in a more rapid decrease in atomic scattering power and, consequently, all of the atomic positions, bond lengths, and bond angles are not sufficiently well determined. Actually, the results closer to the experimental ones were obtained assuming the X-ray structure was determined at 100 K rather than by assuming the X-ray structure at 296 K, as seen in Table 3, although the NQR experiment was performed at 295 K. Moreover, the NQR parameters were reproduced significantly better under the assumption of the optimized structure, which minimizes the influence of the aforementioned error (Table 5). 

Optimization of the low-temperature structures (CCDC 2048895 or 2106318) with fixed unit cell parameters and at the GGA/RBPE level of theory yields more reasonable C–H, O–H, and N–H bond lengths, and amide nitrogen hybridization is a bit closer to the real value (sp^2^). As a consequence, even the asymmetry parameter for –NH_2_ is closer to the experimental value, although still slightly overestimated (by about 20%).

The cluster calculations that take into account all intermolecular interactions only in the immediate vicinity of the molecule, performed with noncovalent forces dedicated to hybrid functional M062X, gave better results. The spread for all nuclei, including –NH_2_, was smaller; however, the NQR parameters were overestimated by an average of 24% and, consequently, the resonance lines were upshifted in Table 6. Such calculations confirmed the validity of the assignment made based on the solid-state calculations and delivered a high-quality function for further QTAIM analysis.

**Figure 5:**
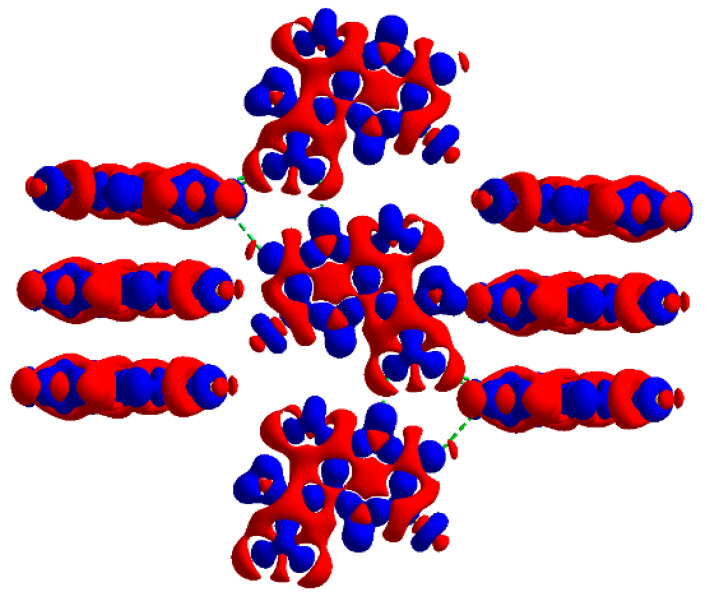
The map of the static deformation density reveals the spatial distribution of the electrons involved in the chemical bonding (isovalue 0.008 a.u.).

It is noteworthy that the assignment of signals to pyrazine nitrogen atoms obtained from the cluster and solid-state calculations are the opposite of those obtained from the single-molecule calculations (Table 3, Table 4, Table 5 and Table 6). Moreover, the same conclusion was drawn regardless of the level of calculations as well as the assumption of each of the X-ray or optimized structures. The detailed analysis of this effect reveals a significant difference between both pyrazine nitrogen atoms. The e^2^Qq/h value of the –N(1)= nitrogen atom increases, while that of the –N(4)= nitrogen decreases, and the decrease in η of –N(4)= is more significant than that of –N(1)=. Moreover, a comparison of the calculated NQR frequencies shows that regardless of the assumed geometry (X-ray or optimized), the changes in the ν_+_ of pyrazine nitrogen –N(4)= and ν_0_ and ν_−_ of amide nitrogen –NH_2_ are the highest. Since ν_0_ and ν_−_ are highly sensitive to hydrogen bonding, the scattering of the two latter clearly indicates the involvement of the –NH_2_ group in such bonds. The map of the static deformation density, Figure 5, reveals the redistribution of valence electron density near nitrogen sites caused by both chemical bonding and intermolecular interactions.

**Figure 6:**
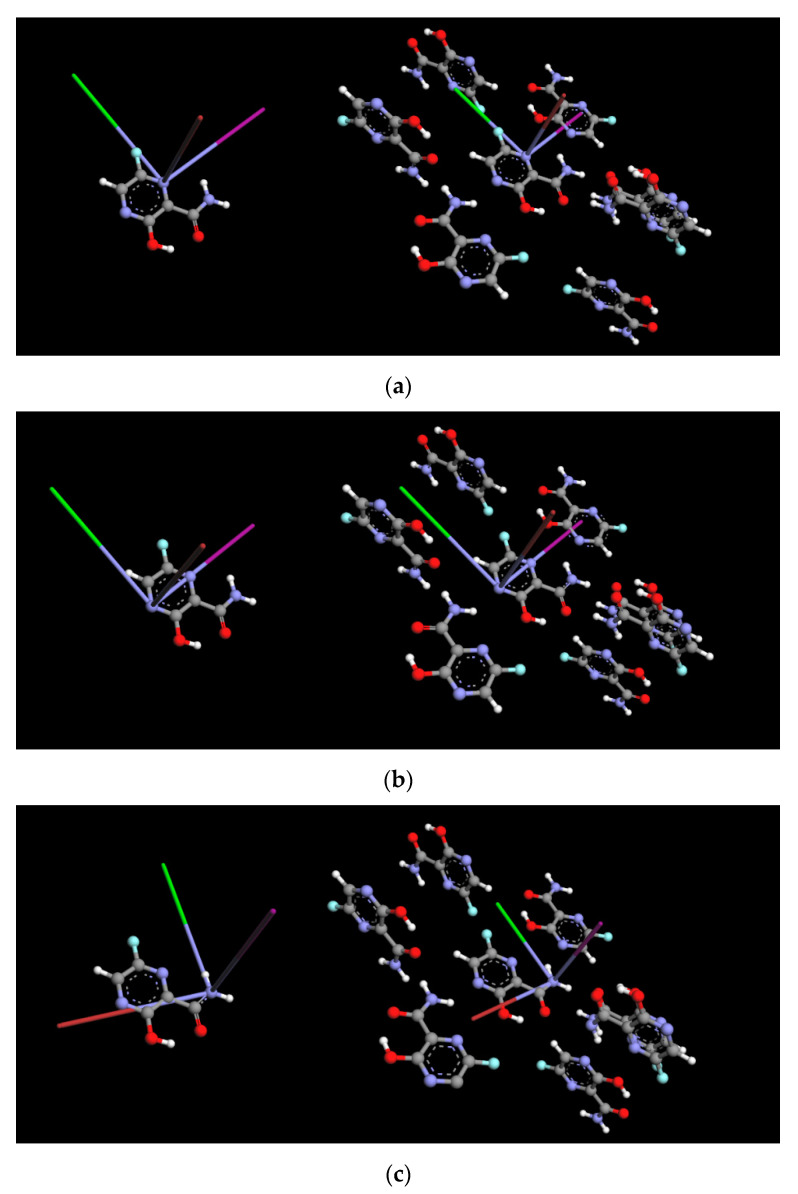
The orientation of the X, Y and Z-axes of the EFG tensor at (**a**) –N(1)=, (**b**) –N(4)=, and (**c**) –NH_2_ (the Z-axis depicted in magenta, the Y-axis in maroon, and the X-axis in green).

The significant charge rearrangement in the solid is concentrated on the atomic nuclei rather than in the internuclear region. The areas of charge accumulation in the bonding regions and the lone-pair positions of the atoms are visible. Upon the N–H···N and N–H···O hydrogen bond formation, the electron density is shifted from the lone-pair orbital of –N= (and =O) to the N–H bond of the acceptor. This effect is reflected in an increase in ν_+_, ν_−,_ e^2^Qqh^−1^, and η of –N(4)=, which is the acceptor, as well as a decrease in ν_−_, e^2^Qqh^−1^, and an increase in ν_+_, ν_0_, and η of –NH_2_, which is the donor. Upon the hydrogen bonding formation, the population of the π orbital of –NH_2_, which is normal to the planar arrangement of N–C and N–H bonds, increases when compared to the populations of the σ orbitals (σ_NC_ and σ_NH_). Therefore, the Z-axis of the EFG tensor is oriented along the π orbital and the X and Y-axes of the EFG tensor are rotated around the Z-axis (Figure 6). 

An increase in σ_NH_, caused by charge transfer from the electron pair of –NH_2_, results in a decrease in e^2^Qq/h. Because σ_NH_ > σ_NC_, the deviation from the axial symmetry along the Z-axis increases, and as a result, η also increases. The fact that e^2^Qq/h is considerably lower for the amide nitrogen in the solid FVP than in a single molecule of FVP supports the above reasoning and is in good agreement with the results obtained, for example, for imidazole. The NQR parameters of –N(4)= are related to the populations of the lone-pair orbital σ_LP_ and the nitrogen orbitals involved in the σ_NC_ and π bonds to carbon. Upon hydrogen bond formation, the X, Y and Z-axes of the EFG tensor at -N(4)= keep their orientation unchanged (Figure 6). However, a decrease in σ_LP_ of –N(4)= results in an increase in e^2^Qq/h and η. The frequencies ν_0_, ν_+_, and ν_−_ are proportional to the populations of the nitrogen atom bonds and both NQR parameters; therefore, this effect results in frequency shifts. The solid-state shift of the NQR parameters for nitrogen –N(1)= is small: η is well reproduced, while e^2^Qq/h is only slightly overestimated. The latter suggests that –N(1)= participates in intermolecular interactions, but not in strong ones. Upon hydrogen bond formation, the X, Y and Z-axes of the EFG tensor at –N(1)= keep their orientation unchanged (Figure 6), which supports this conclusion. However, a possible competition between acceptors (nitrogen, oxygen, and fluorine) raises the question of whether the experimentally observed local nitrogen environment corresponds to the most likely hydrogen bonds, and if the –NH and –OH groups can form intra- and intermolecular interactions.

### 2.2. Interactions Pattern

For a detailed evaluation of the pattern of intermolecular interactions in FVP crystals, we applied 3D HS, 2D molecular fingerprints, and an ESP approach, which helps to identify their type and the QTAIM with RDS for their strength estimation.

#### 2.2.1. Hirshfeld Surfaces

**Figure 7:**
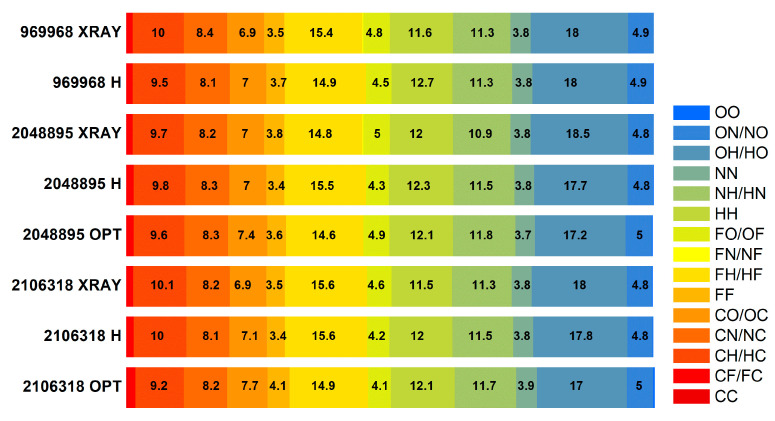
Percentage contributions to the 3D Hirshfeld surface area from various intermolecular contacts.

A combined analysis of 3D Hirshfeld Surfaces (3D HS) with d_norm_, shape index, or curvedness mapped over these surfaces and 2D molecular fingerprints (2D FP) derived from 3D HS, which summarizes the distribution of interactions of a molecule with its environment, delivers an insight into the mix of the 15 types of the homo- and heteronuclear intermolecular interactions in the FVP crystal. As much as 40% of the FVP molecular surface is generated by H atoms (only four), while the other contributing elements C, F, N, and O generate much smaller but quite similar percentages of the molecular surface—from about 13 to 18 % (Table 7 and Figure 7).

Approximately 81% of all interactions of the complex surface consist of seven types O···H, F···H, N···H, H···H, C···H, C···N, and C···O. There are only small differences in the percentages of these interactions for different structures of polymorph I (X-ray, proton, and fully optimized), as shown in Figure 7.

The distribution of interactions is weakly affected by temperature. The whole distribution is, to a small extent, changed by optimization; for heavy atoms within the error limit, by 0.1 %, only for protons, slightly more, by 0.9 %. Thus, the only significant changes after optimization are the positions of the protons, resulting in shorter and less nonlinear and, thus, stronger hydrogen bonds.

The enrichment ratio, E_XY_, of the main intermolecular contacts, which reveals privileged (E_XY_ > 1) and disfavoured (E_XY_ < 1) contacts between every two atomic species, X and Y, are collected in Table 8.

**Figure 8:**
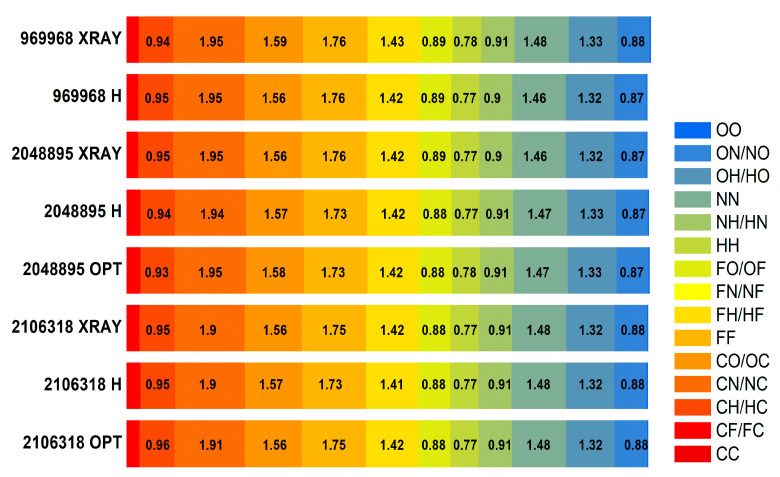
The enrichment ratios from the various contacts (H—protons, OPT—optimized protons).

For all examined FVP crystalline structures of polymorph I (X-ray, proton, and fully optimized), the percentages of the molecular surface and enrichment ratios are very close (the differences do not exceed 2%) (Figure 8).

Four C and two N atoms in the six-member heterocyclic pyrazine ring are exposed to the molecular surface because each of them is three (C) and two (N) bonded (trigonal planar sp^2^ hybridization), and their p orbitals form π bonds above and below the ring. The C···N contacts in FVP are significantly enriched (E_CN_ ≈ 1.9), thus, the C species are the preferred interaction partners for N and more favoured compared to N···N species (E_NN_ ≈ 1.5). However, both contacts have enrichment ratios much higher than unity due to the high nitrogen but low hydrogen abundance in the heterocyclic ring and the high propensity of π stacking with polarised atoms as interacting partners. This suggests that in π⋯π stacking, the C(5)···N(NH_2_), C(3)···N(1), C(6)···N(4) as well as N(1)···N(4) contacts play an important role in the crystalline structure. For the O···C contacts, the enrichment ratio is also high (E_OC_ ≈ 1.5), which suggests that in π⋯π stacking, the O atoms interact with positively charged C atoms, which are themselves covalently bonded to O atoms. Thus, the possibility of the contacts C(6)···O(OH) and C(CONH_2_)···O(OH) is raised. Among C···C and H···C interactions, which compete in the crystal, only C⋯H contacts are favoured, but their enrichment ratio E_CH_ < 1 as H atoms are involved in hydrogen bonds. Concurrently, the C···C contacts are significantly impoverished (E_CC_ ≈ 0), so their participation in π···π stacking is electrostatically unfavourable. A very high E_CH_/E_CC_ suggests the classification of the packing motifs as herringbone [51]. The propensity of F···F and F···H/H···F contacts is significantly enriched (E_FF_ ≈ 2.0 and E_FH_ ≈ 1.4), thus, the F and H species are the interaction partners for F. Among them, the F···F contacts, which form zig-zag chains, are preferred. However, the F···H/H···F contacts are also favoured in the crystal packing and raise the probability of F···HX hydrogen bonds (F···H–C(3) and F···H–O(OH)). The H···H contacts appear with an enrichment ratio significantly smaller than unity (E_HH_ ≈ 0.8) due to their strong competition with both H···F contacts (E_FH_ ≈ 1.4) and the hydrogen acceptors H···N (E_NH_ ≈ 0.8) and H···O (E_OH_ ≈ 1.3), additionally forced by the presence of low hydrogen content. The O···H/H···O contacts show enrichment values (E_OH_ ≈ 1.3) because they are favoured in the crystal packing, and, notably, the O=C oxygen atoms form intramolecular hydrogen bonds of the type O–H···O (closing the five-member ring) and intermolecular bonds of the type N–H···O (–NH from –NH_2_). For the same reason, the O···O contacts are significantly impoverished (E_OO_ ≈ 0.1). The N···H/H···N contacts show enrichment values smaller than unity (E_OH_ ≈ 0.9), and, thus, are disfavoured, and the existence of the N–H···N hydrogen bonds linking –N(4)= and –NH_2_ is not raised. In general, the favoured interaction partners for N, whose contribution to the 3D HS molecular surface is about 16%, are C and N species, while H, followed by O and F are disfavoured.

**Figure 9:**
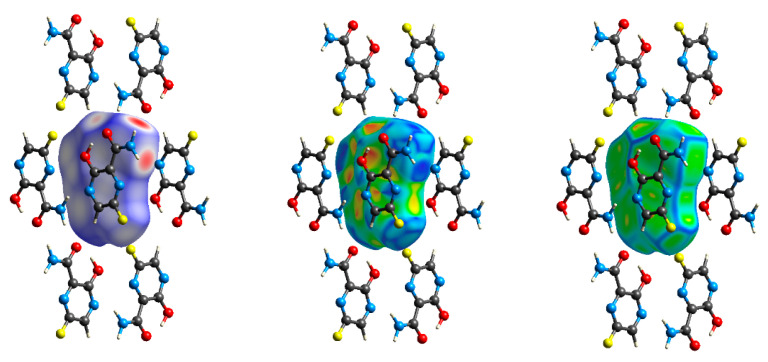
The crystal structure of FVP (2048895, protons optimized), with d_norm_ (**left**), shape index (**centre**), and curvedness (**right**) over a total 3D Hirshfeld surface.

A little more information about the types of interactions and their location is provided by 3D HS, which, with the normalized contact distance d_norm_, shape index and curvedness mapped over this surface, are shown in Figure 9.

The intramolecular O–H···O hydrogen bond is very short (R_O···O_ = 2.592 Å, after optimization even shorter R_O···O_ = 2.503 Å) and nonlinear (<OHO = 144.55°, after optimization more linear <OHO = 154.37°). Intense red areas in the 3D HS near O and H from –OH, represent very low values of d_norm_ = −0.6433 a.u. (after optimization −0.7823 a.u.), shape index = −0.9389 (after optimization −0.8045 a.u.), and curvedness = −1.5591 a.u. (after optimization −1.6515 a.u.), which indicates a strong bond.

The intermolecular hydrogen bonds are indicated in the 3D HS surface by the areas coloured from intense red to white. Intense red areas in the 3D HS localised near =O and H (from –NH_2_) represent the lowest values of d_norm_ = −0.5559 a.u., shape index = −0.9984 a.u., and curvedness = −2.216 a.u., and confirm the presence of a strong N–H···O hydrogen bond, which is short (R_O···N_ = 2.880 Å) and nonlinear (<NHO = 166.95°). The lighter red areas near –N(4)= and H from the –NH_2_ moiety represent the values of d_norm_ =−0.3444 a.u. (shape index = −0.9844 a.u. and curvedness = −1.5983 a.u.) and indicate the presence of a weak N–H···N hydrogen bond, which is long (R_N···N_ = 2.962 Å) and highly nonlinear (<NHN = 133.82°). The light pink areas near =O and H from C(5) represent the values of d_norm_ = −0.8983 a.u. (shape index = 0.9512 a.u. and curvedness = −2.2851 a.u.). They imply the presence of a weak C(5) –H···O hydrogen bond, which is long (R_C···O_ = 3.421 Å) and nonlinear (<CHO = 148.91°). The white pink areas near F and H from C(5) represent the values of d_norm_ = 0.219 a.u., shape index 0.751 a.u., and curvedness= −1.635 a.u., and indicate a weak C–H···F hydrogen bond of 3.445 Å and <118.9°.

The abovementioned four types of bonds are competitive to van der Waals contacts of 3.068 Å linking F···F, which are shown as the white areas in the d_norm_ surface, with d_norm_ = 0.0915 a.u, shape index = 0.6253 a.u., and curvedness = −1.6668 a.u. The bright-yellow area in the curvedness surface also suggests the presence of F···O (O from –OH) contact of 3.831 Å characterized by values of d_norm_ = 0.1891 a.u., shape index = 0.0235 a.u., and curvedness = −2.4213 a.u., and N(1)···O (O from –OH) contact of 3.379 Å, with the values of d_norm_ =0.2130 a.u., shape index = −0.4859 a.u., and curvedness = −1.6035 a.u. The N···C contacts of 3.350–3.574 Å are described by the values of d_norm_ = 0.087 a.u., shape index = −0.704 a.u., and curvedness = −1.841 a.u.

Optimization of the geometry with fixed parameters of the unit cell results in the shortening of hydrogen bonds in relation to crystallographic bonds, which affects the 3D crystal pattern. This effect is poorly reflected by the shape of the 3D HS surface but well described by d_norm_, shape index, and curvedness. The N–H···O is described by values of d_norm_ = −0.6313 a.u., shape index = −0.9428 a.u., and curvedness = −3.3128 a.u., while the N–H···N is characterized by values of d_norm_ = −0.4436 a.u., shape index = −0.9957 a.u., and curvedness = −1.4333 a.u. Both are shorter (R_O···N_ = 2.796 Å and R_N···N_ = 2.915 Å) and more linear (<NHO = 170.87° and <NHN = 142.20°), and, thus, stronger. The C–H···O hydrogen bond, which is described by values of d_norm_ = −0.1367 a.u., shape index = 0.8807 a.u., and curvedness = −1.8613 a.u. is longer (R_C···O_ = 3.542 Å) and weaker. The C(5) –H···F hydrogen bond of length 3.542 Å is characterized by values of d_norm_ = 0.2989 a.u., shape index = 0.7926 a.u., and curvedness = −1.5158 a.u., i.e., weaker. The N···C contacts of 3.351–3.411 Å are described by values of d_norm_ = 0.068 a.u., shape index = −0.629 a.u., and curvedness = −1.738 a.u.

**Figure 10:**
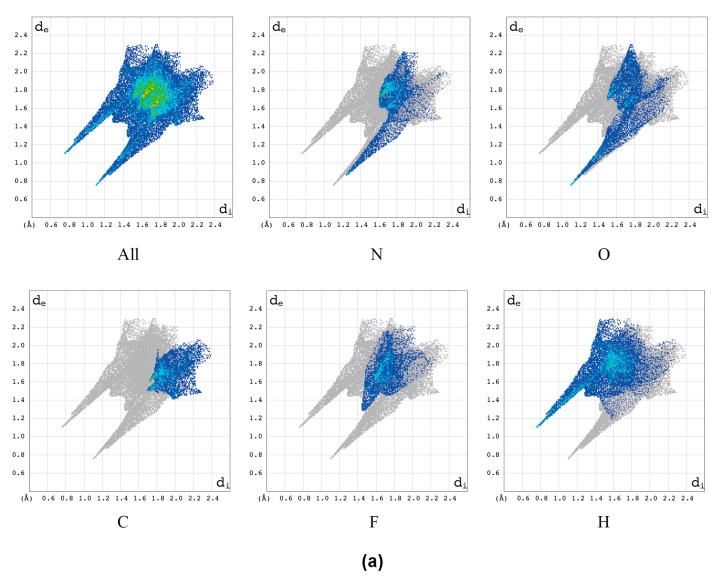

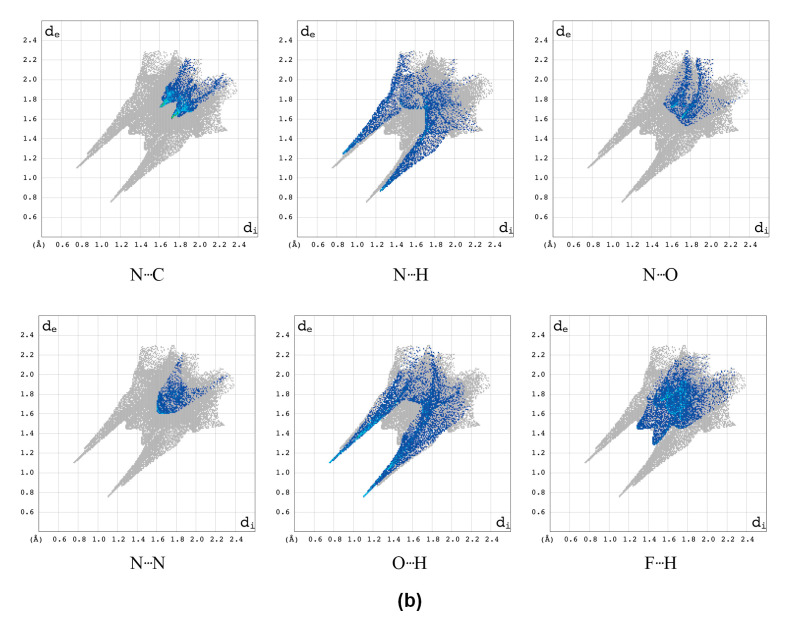
2D molecular fingerprints of the interactions pattern in FVP (2048895, protons optimized) and its breakdown to the (**a**) particular species (N, O, C, F, and H) and (**b**) selected contacts (N···C, N···H, N···O, N···N, and F···H).

In order to verify the presence of interactions, mainly directional interactions, the characteristic features of the 2D fingerprint (2D FP) *d*_e_/*d*_i_ plots (Figure 10), obtained from the 3D HS, were further analysed.

In the 2D FP plot, the O···H/H···O contacts describing intramolecular O–H···O and intermolecular N–H···O hydrogen bonds are represented by symmetric spikes (‘‘wings’’), which are sharp and small, at *d*_e_ + *d*_i_ ~ 2.0 Å (*d*_e_ + *d*_i_ ~ 1.75 Å for the optimized geometry). These interactions cover a small area of 17.8% (17% for the optimized geometry) of the total 3D HS. The N···H/H···N contacts are represented by the most external symmetric spikes sharp, and *d*_e_ + *d*_i_ ~ 2.2 Å (*d*_e_ + *d*_i_ ~ 2.0 Å for the optimized geometry). These interactions cover a small area of 11.5% (11.7% for the optimized geometry) of the total 3D HS. The F···H/H···F contacts are represented in 2D FP by the widest spikes at *d*_e_ + *d*_i_ ~ 3.00 Å. They cover 15.6% (14.9% for the optimized geometry) of the total 3D HS. The C···O/O···C contacts bring a contribution of 7.1% (7.7% for the optimized geometry) and they are represented by sharp wings, *d*_e_ + *d*_i_ ~ 3.00 Å (3.10 Å for the optimized geometry), located in the middle area of the whole fingerprint. The O···N, O···F, and F···F contacts bring a small contribution of 4.8, 4.2, and 3.4% (5.0, 4.3, and 4.1% for the optimized geometry), respectively. The relatively high contribution is brought by weak H···H interactions, which cover 12.0 % (12.1% for the optimized geometry) of the total 3D HS. In the 2D FP, they are reflected by the cloud of scattered points. The contribution brought by C···N/N···C and N⋯N contacts, which cover 8.1 and 3.8 % (8.1 and 3.9% for the optimized geometry), respectively, mainly comes from C(3)⋯N(4) and N(1)···N(4) contacts, i.e., interlayer π···π stacking interactions.

**Figure 11:**
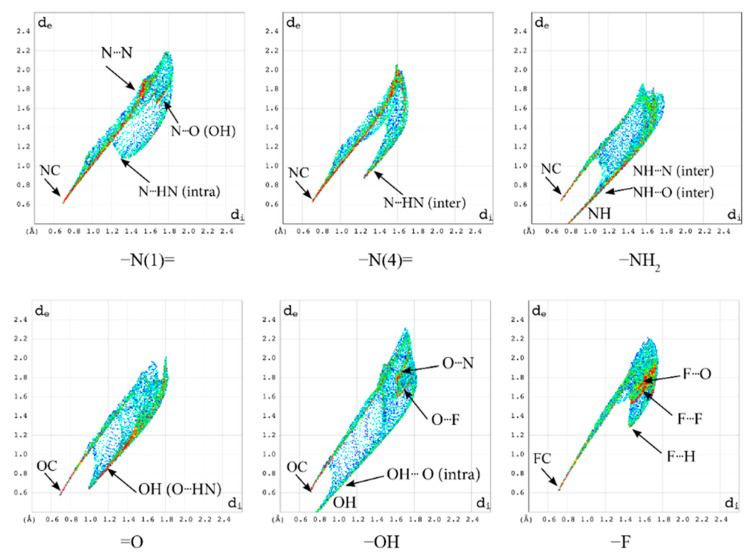
Local 2D molecular fingerprinting of the interactions pattern in FVP (2048895, protons optimized) for –N(1)=, –N(4)=, –NH_2_, =O, –OH, and –F.

The local 2D molecular fingerprints of –N(1)=, –N(4)=, and –NH_2_=, generated based on 3D HS for the particular nitrogen atoms (Figure 11), vary significantly from one nitrogen site to another, in both shape and percentage.

While the upper narrow wing attributed to the NC covalent bond is common and featureless, the lower one matters because it shows a considerable variation in the number (three in –N(4)=, four in –N(1)=, and two in –NH_2_) and type of interactions.

The local 2D FPs reveal the participation of –NH_2_ in two covalent NH bonds being donors for two strong hydrogen bonds NH···O and NH···N and the participation of –N(4)= in intermolecular N–H···N hydrogen bond and N···N, N···O contacts, Table 9.

Moreover, it suggests the involvement of –N(1)= in the intramolecular hydrogen bond N–H···N (which closes the five-member ring but was not detected in the global 3D HS-based analyses) and in three N···N, N···O, and N···F contacts. The wing describing the intramolecular hydrogen bond N–H···N, very wide and scattered at *d*_e_ + *d*_i_ =2.4 Å, is highly different from the intermolecular NH···N, which is very narrow and at *d*_e_ + *d*_i_ = 2.15 Å. A similar analysis of the local 2D FP for the oxygen atoms reveals a sharp upper wing attributed to the OC covalent bond at *d*_e_ + *d*_i_ = 1.3 Å and a bottom wider wing attributed to hydrogen bonds (intermolecular O–H···N at *d*_e_ + *d*_i_ = 1.65 Å for =O and intramolecular O–H···O at *d*_e_ + *d*_i_ = 2.15 Å for –OH) and some heavy-atom contacts, Table 9.

The wing describing the intramolecular hydrogen bond O–H···O is typical of this kind of bond. It is worth noting that N–H···N has a very similar shape of a wide and scattered cloud of points. The local 2D FP of the fluorine atom reveals a sharp upper wing attributed to the FC covalent bond and a wide bottom one indicating the involvement of fluorine in the F···H–C hydrogen bond and three kinds of contacts F···N, F···O, and F···F (Table 9).

Thus, the analysis of local, atomic, 2D molecular fingerprints helps to discover/confirm the presence of the very weak hydrogen bonds N···H–N (intramolecular) and F···H–C (intermolecular) which meet the Gilli criterion [52] and are nicely complementary to the total 3D HS and 2D FP. Moreover, they explain the source of the differences between particular nitrogen nuclei detected by NQR.

#### 2.2.2. Characterization of the Strength of the Interactions

##### The Total Interaction Energy Partitioning

**Figure 12:**
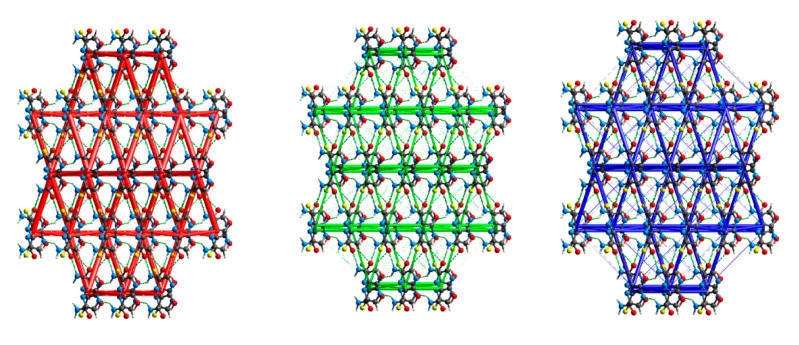

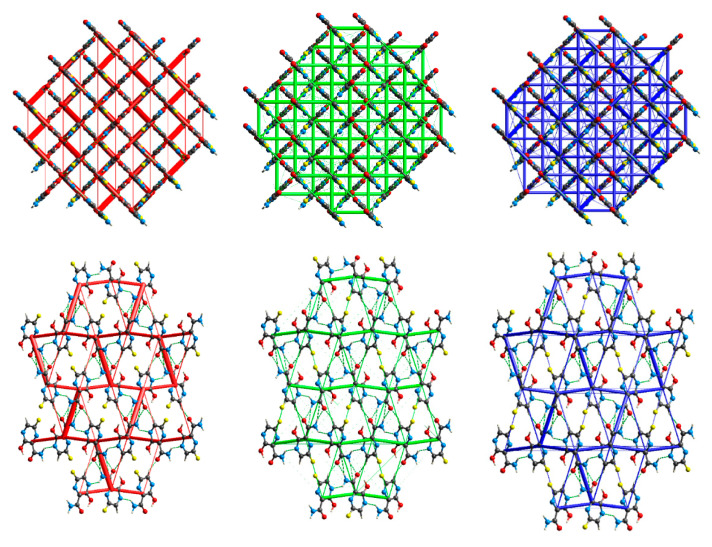
Energy framework of diagrams for electrostatic, dispersion, and total energy visualised in the FVP structure by red, green, and blue “sticks”, respectively (view along the a-axis—(**top**), along the b-axis—(**middle**), and along the c-axis—(**bottom**)).

The analysis of the total lattice energy and its terms (electrostatic, polarization, dispersion, and repulsion) derived from DFT calculations for a cluster of 10 Å in diameter has revealed that irrespective of temperature, the major contributions come from the electrostatic or dispersion terms and could not be cancelled by the repulsion. The total lattice energy (−105.6 kJ/mol) is dominated by the dispersion (−77.17 kJ/mol) and electrostatic (−70.82 kJ/mol) terms, which are stronger than the polarization (−13.76 kJ/mol) or repulsion terms (56.79 kJ/mol). Upon a temperature increase, all negative terms increase dispersion (−72.82 kJ/mol), electrostatic (−63.21 kJ/mol), and polarization (−12.14 kJ/mol), while repulsion decreases (48.32 kJ/mol), which results in slightly lower total energy (−100.5 kJ/mol). The optimization with the fixed unit cell results in an increase in each term of energy (electrostatic (−78.32 kJ/mol), dispersion (−77.95 kJ/mol), polarization (−15.98 kJ/mol), and repulsion (65.26 kJ/mol), which in turn causes a decrease in the total energy (−95.2 kJ/mol). The total interaction energy partitioning indicates that the crystalline packing is determined by mainly electrostatic and repulsive strong N–H⋯O hydrogen bonds followed by N−H···N and weaker C–H⋯O, and dispersive strong π⋯π stacking and relatively weak F⋯F contacts (Table 10 and Figure 12). (However, a partial contribution to the repulsion term in N–H⋯N may, in fact, come from N⋯C contacts).

The ordering of the most important interactions according to the decreasing energy is as follows:

N–H⋯O ≈ π⋯π stacking (parallelly oriented molecules) >

>N–H⋯N ≈ π⋯π stacking (flipped molecules) > C–H⋯O >> F⋯F.

The N–H⋯O hydrogen bonds and π⋯π stacking (slightly different between parallel or flipped molecules) forcing planar arrangement and F⋯F contacts forming zig-zag chains, are the main driving forces acting cooperatively (Figure 12).

##### Electrostatic Potential

**Figure 13:**
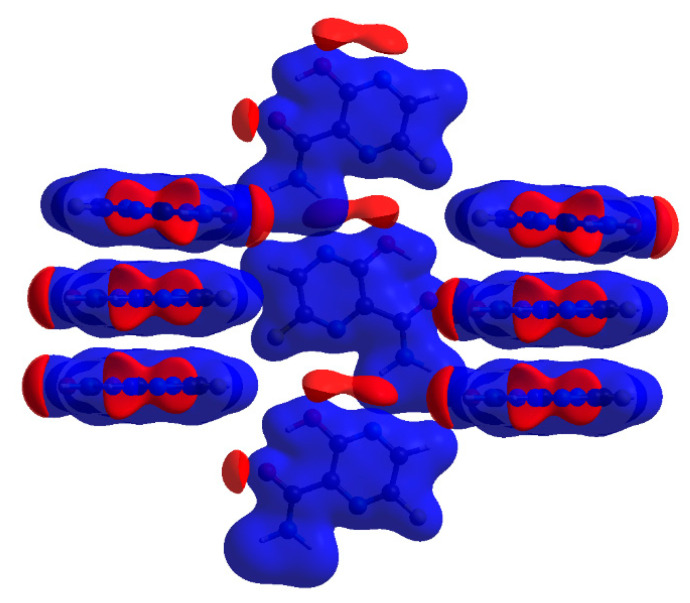
Electrostatic complementarity upon crystal packing in FVP: electrostatic potential mapped onto the Hirshfeld surfaces of symmetry-related neighbouring molecules. The colour scale is −0.05 (red) to 0 (white) to 0.05 a.u. (blue).

The electrostatic nature of the hydrogen bonds, that is, the match of positive and negative regions of the molecular surfaces (electrostatic complementarity), is also well pronounced in the molecular electrostatic potential (ESP). The positive (blue) regions of ESP are clearly separated from the negative (red) regions in Figure 13.

The negative ESP on both sides of the molecule occupies O and double O infinity sign and hood-shaped regions. The highly positive region is localised on almost the whole moiety, including –NH_2_ and –N(1)=, while the negative one is only on nitrogen –N(4)= and both oxygens. The arrangement of the positive and negative ESP sites helps to understand hydrogen bond formation. The negatively charged regions located at –N(4)=, –OH, and =O are linked by bonds to the positively charged regions located at –NH_2_, –N(1)=, and –C(5)H (they perfectly match each other). As a result, N-H⋯O and C-H⋯O hydrogen bonds and N(4)⋯O contacts are formed. Moreover, the N–H⋯N hydrogen bond, although dominated by electrostatic forces, is not pronounced in the ESP due to the lack of electronegativity difference between the acceptor and donor. These observations are extremely important from the docking point of view as –N(4)= plays a key role in the prodrug conversion to the active substance (phosphoribosylation).

##### Quantum Theory of Atoms in Molecules

**Figure 14:**
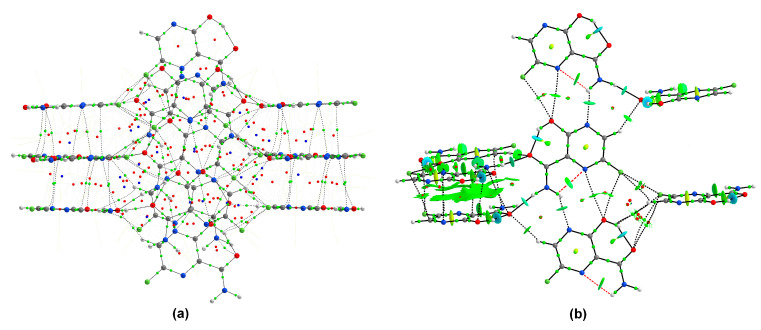
Molecular graph of FVP molecule with (**a**) all nearest neighbours within a 3.8 Å radius and (**b**) selected neighbours cut off from the crystalline structure (BCP—small green points, RCP—small red points). The isosurface of the reduced density gradient surface (RDG = 0.5) with sign(λ_2_)ρ_BCP_ is mapped over the surface. The colour scale is −0.07 (blue) to 0.07 a.u. (red). A very weak putative intramolecular hydrogen bond N–H···N not detected by QTAIM but indicated by the disc-shaped green surface of the RDG located between the –N(1)= and H (from –NH_2_) is depicted in red dotted line.

The quantum theory of atoms in molecules (QTAIM) [31] approach was used for the verification and strength estimation of the inter- and intramolecular interactions revealed by 3D HS and 2D FP. The topological descriptors of electron density (ρ(r) and Δρ(r), the H_BCP_(r) and its components G_BCP_(r) and V_BCP_(r)), as well as the strength of all intra- and intermolecular bonds and contacts, are collected in Table 11. In general, QTAIM is, in some cases, not sufficient to detect weak noncovalent interactions [32], thus the isosurface of the reduced density gradient RDG(r) was examined, and the corresponding plot was generated for the FVP structure. The molecular graphs cut off the crystalline structure with BCP, RCP, and reduced density gradient isosurfaces (RDG) with sign(λ_2_)ρ_BCP_ mapped over the surface, as shown in Figure 14.

**Figure 15:**
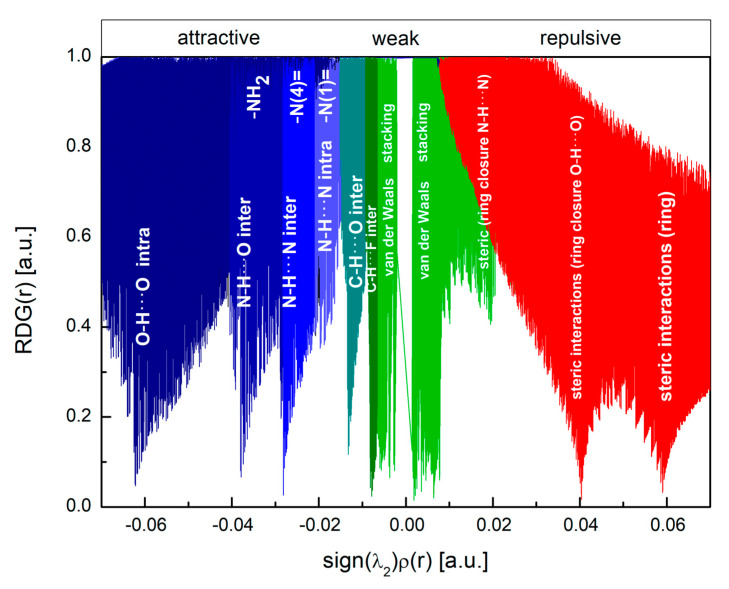
A 2D plot of RDS(r) vs. sign(λ_2_)ρ(r) revealing spikes describing the weak non-covalent interactions of hydrogen bonds, van der Waals contacts, and steric interactions in the FVP crystal.

Results of QTAIM confirmed the presence of the hydrogen bonds and contacts detected by the total 2D FP. The energies estimated using QTAIM and Equations (7)–(12), Table 11, are in good agreement with those estimated using the energy framework. In contrast to the local 2D FP, QTAIM did not detect intramolecular N–H···N hydrogen bonds. However, the disc-shaped green surface of the reduced density gradient located between the –N(1)= and H (from –NH_2_) indicated the presence of a weak intramolecular N–H···N hydrogen bond, although no critical points were detected (Figure 14). In the plot of RDG(r) versus sign(λ_2_)ρ(r), Figure 15, the noncovalent interactions—hydrogen bonds (strong and attractive), van der Waals contacts (weak), and steric interactions (ring closures, repulsive)—are visible as spikes.

Their positions were identified based on the analysis of the closely related pyrazine structures. The intramolecular N–H···N hydrogen bond spike is shown in light blue. The analysis of the bonds in which each of the nitrogen atoms participates within QTAIM and RDG reveals significant disproportions between them. The –NH_2_ site participates in two intermolecular hydrogen bonds of different strengths N–H···N and N–H···O; –N(4)= participates in N–H···N intermolecular hydrogen bond, but –N(1)= only in a very weak putative intramolecular hydrogen bond N–H···N. This is reflected in the asymmetry parameters, the highest for asymmetrically bonded –NH_2_, and the lowest for –N(4)=, participating only in one bond coaxially with the Z-axis of the EFG tensor. The position of the spike in the plot of RDG(r) versus sign(λ_2_)ρ(r), Figure 15, is very well correlated (85%) with the e^2^qQ/h.

### 2.3. Binding Mode of Biologically Active Form

For FVP to be biologically active, it must undergo intracellular phosphorylation to its active form (FPV triphosphate). A false nucleoside, which is built by the viral RdRP into the nascent viral RNA, results in a “defective”, mutated RNA [17,18]. Three structures of COVID-19 RNA-dependent RNA polymerase bound to FVP (7DFG [53]), Nsp7-Nsp8-Nsp12 SARS-CoV-2 RNA-dependent RNA polymerase in complex with template primer dsRNA, and FVP-RTP (7AAP [54]), and replicating polymerase complex of SARS-CoV-2 in the precatalytic state bound to FVP (7CTT [55]), were retrieved from the PDB database.

In 7DFG, the FVP moiety has adopted the same amide group conformation as in the solid state, while in 7AAP and 7CTT, the amide group of FVP-RTP is rotated by 180° around the -C(2)-CONH_2_ bond, although it is energetically unfavoured (by 5.8 kJ/mol). Consequently, the two types of structures differ significantly in the binding mode within the FVP moiety. In the 7DFG structure, FVP mimics guanosine, and through its amide group, forms a noncanonical base pair (the so-called wobble base pair) with uracil in the template RNA strand bonded via two intermolecular N–H⋯O hydrogen bonds of 2.452 Å and 2.268 Å. Two O–H⋯O hydrogen bonds of 2.909 Å (10.46 kJ/mol) and 2.939 Å (10.02 kJ/mol) link =O from FVP with SER682. The intramolecular hydrogen bond N–H⋯N of 2.711 Å closes a 6-membered ring, while the π⋯π stacking between nucleoside pairs (uracil⋯FVP and adenosine⋯uracil), where an amide group links N(1) with adenosine and C(5) with uracil, additionally stabilize the structure. In the 7AAP structure, FVP mimics the guanosine base, and through its amide group, forms a noncanonical base pair with cytosine in the template RNA strand. Three intermolecular hydrogen bonds, N–H⋯O of 2.569 Å (=O from FVP), N–H⋯O of 3.170 Å, and N–H⋯N of 2.740 Å (–NH_2_ from FVP) bind the cytosine, and two other bonds, strong N–H⋯O (–NH from FVP) of 2.989 Å and weak C–H⋯O (=O from FVP) of 3.539 Å, bind SER682. This system is additionally stabilised by an intramolecular hydrogen bond, N–H⋯O, of 2.549 Å (linking =O with –NH_2_) closing a 6-member ring. Moreover, the van der Waals-type side chain contact C⋯O of 3.24 Å and the hydrogen bond N–H⋯O of 3.28 Å (involving =O with C(6) and N(6) from adenosine) supports the π⋯π stacking interaction realised by C(5) of FVP and uracil (average stack distance 4.06 Å) and the N⋯N contact (linking –N(1)= of FVP and N(3) of uracil) of 3.22 Å.

In the 7CTT structure, two N–H⋯O hydrogen bonds of 2.4 Å and 3.2 Å and N–H⋯N of 2.74 Å, involving amide group through =O and –NH_2_, respectively, link the FVP moiety with cytosine in the template RNA. Two O–H⋯O hydrogen bonds of 2.44 Å and 3.38 Å link =O from FVP and one N–H⋯O of 2.99 Å links –NH_2_ from FVP with SER682. This system is additionally stabilised by an intramolecular N–H⋯O hydrogen bond of 2.612 Å, closing a 6-member ring. The nitrogen atom from the –NH_2_ group forms the π⋯π stacking with uracil (average stack distance 3.97 Å), while C(5) forms π⋯π with adenosine (average stack distance 4.32 Å). In addition to base pairing with cytosine or uracil, FVP is coordinated by Lys545, which is positioned to accept hydrogen bonds from the nitrogen -N1= in the pyrazine ring or is less likely to donate to the fluorine atom (3.4 Å and 3.7 Å or 3.6 Å, and 4.6 Å for cytosine or uracil base pairing, respectively). While in the solid F···H, contacts are preferred, and weak C-H···F bonds are present in FVP-RTP bound with the RNA strand, which fluorine cannot form because the nearest protons are too far. Fluorine also does not appear to be directly involved in the base pairing but still plays a role in the binding as it makes contact with neighbouring N, C, or O atoms in the stack. In 7DEF, it participates in the F⋯O contact of 3.321 Å and supports the C–H⋯O hydrogen bond of 3.065 Å, both linking the FVP moiety with the ribosyl ring and forcing specific conformation of RMP. Additionally, F⋯C (C from uracil) of 3.544 Å supports the π⋯π stacking. In 7AAP, fluorine participates only in the F⋯O contact of 3.674 Å, but supports the C–H⋯O hydrogen bond of 3.082 Å forcing the specific RTP conformation. In 7CCT, it participates in two F⋯O contacts of 4.957 Å and 4.691 Å, supports the C–H⋯O hydrogen bond of 2.694 Å to keep the specific RTP conformation, and, additionally, supports the π⋯π stacking by F⋯N (N from adenosine) of 4.151 Å. All of this fits the typical pattern of fluorine’s role within drug structures, i.e., the impact on conformation.

Moreover, the FVP-RMP (in 7DFG), FVP-RTP (in 7AAP; active state) and (in 7CTT; precatalytic state) also differ in the conformation of RMP and RTP, which are substituted at the –N(4)= position of FVP. As a consequence, in the former, RMP binds with only 5 residues (ASP623, ASP 760, U20, and magnesium ion), while in the remaining two, RTP adopted two different conformations, which results in binding with 7 (ASN691, ASP623, ASP760, U20, SER814, and two magnesium ions) and 11 (ASP623, ASN691, THR687, ARG555, LYS798, LYS621, ARG553, ASP618, ASP760, A20, SER682, and one magnesium ion) residues, respectively. The active site in RdRp polymerase, a principal target for the SARS-CoV-2, is known to be formed by two catalytic motifs: A, composed of the residues from 611 to 626 and C, which contains residues from 753 to 767 [56]. The most conserved residue in viral RdRp is the classic divalent-cation-binding ASP618, while the critical catalytic residues, responsible for RdRp activity, are SER759, ASP760, and ASP761. Multisubunit RNA polymerase binds nucleotide triphosphate (NTP) substrates, which enter the main-enzyme channel via a hydrophilic cluster containing LYS545, ARG553, and ARG555 [56].

The FVP-RMP (in 7DFG) interacts noncovalently with ASP618 (5.44 kJ/mol), SER759 (−3.93 kJ/mol), ASP760 (−4.52 kJ/mol), and ASP761 (5.52 kJ/mol), as well as LYS545 (−7.49 kJ/mol), ARG553 (−4.27 kJ/mol), and ARG555 (−5.73 kJ/mol), but the main target residues are SER682 (−55.40 kJ/mol), ASN691 (−17.62 kJ/mol), and ASP623 (−13.77 kJ/mol). However, its binding to the Mg cofactor is very strong (−91.59 kJ/mol). After phosphorylation, the ligand’s binding strength increases significantly. Although FVP-RTP (in 7CTT; precatalytic state) interact with conserved residue ASP618 (61.80 kJ/mol) and three critical residues SER759 (−3.10 kJ/mol), ASP760 (10.96 kJ/mol), and ASP761 (19.50 kJ/mol), these interactions are primarily electrostatic and repulsive. The bindings of FVP-RTP with LYS798 (−99.24 kJ/mol), LYS621 (−86.44 kJ/mol), SER682 (−28.74 kJ/mol), ASP623 (−17.03 kJ/mol), and ASN691 (−19.41 kJ/mol) are significantly stronger and attractive. Its binding to the hydrophilic cluster residues LYS545 (−22.51 kJ/mol), ARG553 (−62.97 kJ/mol), and ARG555 (−130.92 kJ/mol) is very strong, but its binding to the Mg cofactor is weak, only −29.37 kJ/mol. 

Similarly, FVP-RTP (in 7AAP; active state) interacts weakly with ASP618 (21.92 kJ/mol), SER759 (−3.31 kJ/mol), ASP760 (7.45 kJ/mol), and ASP761 (25.82 kJ/mol), but binds strongly with LYS545 (−39.46 kJ/mol), ARG553 (−19.33 kJ/mol), and ARG555 (−33.77 kJ/mol). Its interactions with two Mg cofactors are very strong (−119.54 and −184.51 kJ/mol), which indicates the high stability of the protein–ligand complex. 

Considering the cofactor binding strength, the ligands can be ordered as FVP-RTP (active state) > FVP-RTP (precatalytic state)>FVP-RMP. The total binding affinity (between target protein and ligand) is also highest in 7AAP (−494.30 kJ/mol), followed by 7CTT (−471.58 kJ/mol), and 7DEF (−247.02 kJ/mol). However, the conformational change in RTP (precatalytic vs. active) may be dictated not only by its binding to the cofactor but may also result from an allosteric effect. 

The allosteric sites in 7AAP were detected with Allosite-Pro [57] and PASSer [58] using a scheme [59,60]. Near the active site, there are two allosteric pockets on either side of the RNA strand. One of the pockets is composed of VAL557, ARG555, LYS545, THR680, SER682, THR556, ASP623, C10, THR687, SER759, ASN691, ALA688, ASP760, ARG553, A11, and U20, and, thus, includes critical catalytic and hydrophilic cluster residues. The procedure used to dock the FVP-RTP ligand in the protein pocket was identical to that previously described [61,62]. The search space was defined as a subset region of 9.0–15.0 Å around the allosteric site. After docking, the best pose that leads to the stabilization of the complex with the highest binding/docking score was selected. 

Docking of the FVP-RTP directly into this pocket leads to its very strong bindings to hydrophilic cluster residues ARG555 (−86.32 kJ/mol), ARG553 (−70.46 kJ/mol), and LYS545 (−41.71 kJ/mol), conserved residues ASP623 (−69.16 kJ/mol) and ASP618 (20.84 kJ/mol), critical catalytic residues ASP761 (21.38 kJ/mol; repulsion), and two Mg cofactors (−216.38 and −215.77 kJ/mol). The total binding affinity is −402.96 kJ/mol. The conformation of FVP-RTP is very similar to that of the RNA template (root-mean-square deviation of atomic positions, RMSD, is only 4.98%). The contribution of the cofactor–ligand electrostatic interaction to the total protein–ligand binding is almost twice as high as the sum of the steric and hydrogen bond contributions. Such a strong direct binding of FVP-RTP to the active site and cofactor suggests a possible alternative mechanism of FVP action, which may explain the scattering of the results of clinical trials [25,63] or the synergistic effect observed in combined treatment against SARS-CoV-2 [64].

**Figure 16:**
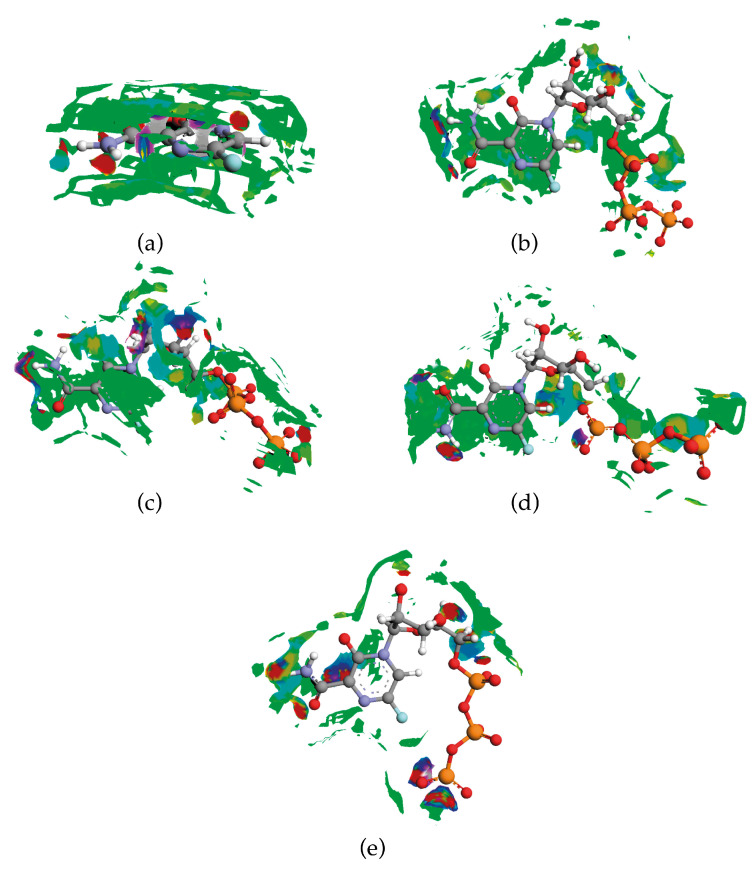
The isosurface of RDG (isovalue 0.5 a.u.) with sign(λ_2_)ρ_BCP_ mapped over the surface revealing binding modes in (**a**) crystal, (**b**) 7AAP, (**c**) 7CTT, (**d**) 7DEF and (**e**) allosteric pocket (for clarity, only single-molecule structures are shown). The colour scale is from −0.07 (red) to 0.07 a.u. (violet).

The binding modes calculated for the nearest vicinity of the single FVP molecule in crystal and FVP-RMP or FVP-RTP molecules in proteins are shown in Figure 16. For clarity, only single-molecule structures are shown.

There is one more nonobvious difference between the solid and RMP/RTP, precatalytic/active states. The intramolecular hydrogen bond O–H⋯O observed in the solid FVP does not occur in any substituted form. However, the weak intramolecular hydrogen bond N–H⋯N is still present in the 7DEF and is replaced by a much stronger N–H⋯O of 2.55 Å and 2.61 Å in active 7AAP and precatalytic 7CTT, respectively. Thanks to the N–H⋯O intramolecular bond, FVP-RTP successfully mimics guanosine. However, this bond strongly affects the intermolecular binding capacity of –NH_2_ and =O. In any case, the intramolecular bonds and, consequently, the planar conformation of the FVP moiety, is not only solid-specific but is also retained in solution during the interaction of the molecule with RNA protein. Furthermore, unlike the solid state with the enol-like conformation of the amide, each active/bonded form of FVP is deprived of a proton at –OH, which implies a keto-like conformation of the amide. Thus, the acceptor–donor balance is shifted in favour of the acceptors. The hydrogen bond acceptors (two O, N, and not fulfilling this role, F), which exceed the hydrogen bond donors (–NH_2_) in number, result in a more polar (thus easily soluble in water) compound. This, in turn, increases secondary electrostatic interactions characterized by no transfer or the sharing of electrons. The latter is an important factor for water-mediated hydrogen bonds that affect protein–RNA recognition. The binding modes described above support the assumption that FVP stops replication through noncovalent interactions in the active site and also suggest that the binding strength, and thus efficiency, may strongly depend on the conformation of the amide and triphosphate groups as well as the acceptor–donor balance. This may explain why the results of clinical trials in the treatment of COVID-19 with FVP have been so conflicting so far [25,63]. Changing the substituents to maintain the preferred conformation of the solid amide and triphosphate groups but also the number of acceptors seem to be good steps in the direction of better efficiency of this drug.

The intrinsic bond strength index [65], a new feature describing the degree of electron density sharing between the atom and its surrounding, DOI, calculated for single FVP and FVP-RTP molecules, partly supports the above observations. The highest DOI values were found for the acceptors –F (37.62%), followed by –C(6) (41.38%), –N(1)= (5.44%), and –N(4)= (1.78%). The lowest DOI were obtained for –NH_2_ (0.38%), –OH (0.32%) acting as a donor, and =O (0.13%). The values of DOI for the conformation flipped by 180° (nonsolid one) are only slightly different. They are the highest for F (37.91%), followed by –C(6) (42.12%), –N(1)= (5.47%), and –N(4)= 1.83%. The donor sites –NH_2_ (0.29%) and –OH (from amide; 0.32%) as well as acceptor =O (0.35%) have very low DOIs. The differences caused by the flip of the amide group are, therefore, negligibly small. In contrast, the ribosylation and phosphorylation significantly change the DOI atomic values. The highest values of DOI for FVP-RMP are shifted from the FVP moiety to ribosyl, for C(1) (29.7%), followed by C(2) (12.7 %), C(3) (12.28%), and O(15) (9.5%), which is independent of the RTP conformation. This reflects the loss of the F’s ability to share electron density with its surroundings in favour of RTP and the tendency of RTP to form multiple bonds evident in both structures. In RMP, the highest DOI are obtained for phosphoryl moiety atoms (32.4% for O(3), 34.78% for P, 4.9 for O(2), 4.33 for O(3), and 4.34% for O(4)), which reflects its ability to bind phosphate groups. Very low DOIs in the –NH_2_ group indicate its strong donor properties, irrespective of the structure of FVP, FVP-RTP, or FVP-RMP. Thus, the –NH_2_ group, which links neighbouring molecules in crystals and base pairs in the RNA template, mainly by strong intermolecular N–H⋯O bonds, plays a key role in both the crystal and protein structure. Its involvement in strong intramolecular N–H⋯O hydrogen bonds seems to be an advantage when it comes to planarity and mimicking guanosine, but it may be an obstacle that weakens the bond pairing. Moreover, these results are in very good agreement with the docking results, which indicate that the phosphoryl oxygens bind to the cofactor.

## 3. Material

The identity and purity of the active substance sample manufactured in China by Jiangsu Zenji Pharmaceuticals Ltd. have been checked in an independent laboratory with NMR and LC–MS methods, and it does not raise any doubts. The purity of the powdered sample was confirmed to be above 98%. The experimental study was performed using the FPV sample without prior recrystallization or any additional purification. The powdered sample was degassed and sealed in a glass ampoule.

## 4. Methods

### 4.1. Experimental—^1^H-^14^N NQDR

The ^14^N NQR frequencies in FVP lie in the low-frequency range below 4 MHz, and direct NQR measurement on a small sample (approx. 0.5 g) is not possible due to a very low signal-to-noise ratio. Therefore, the multistep procedure was used. The ^14^N NQR frequencies were determined indirectly by measuring the proton NMR signal using three nuclear quadrupole double resonance techniques: ^1^H-^14^N cross-relaxation (CR) [39], multiple frequency sweeps, and two-frequency irradiation [42,43].

### 4.2. Spectra Simulations—Density Functional Theory (DFT)

Quantum chemical calculations were carried out within the density functional theory (DFT) approach rooted in Kohn–Sham’s [66] theorem, generalised by Levy [67]. The Minnesota hybrid meta M062X, high-nonlocality exchange-correlation functional with double the amount of nonlocal exchange (2X), [68] and all-electron split-valence basis set 6 – 311 + G(d,p) were applied for the single molecule and cluster calculations. The advantage of M062X with 54% HF exchange is that it gives good results for the system with noncovalent forces, which is one of the biggest deficiencies of standard DFT methods. Because the X-ray crystallography at the usual resolution often fails to directly access the positions of light atoms, the proton positions are poorly identified. Therefore, the corrected (optimized) proton positions are routinely used by us in all analyses based on the electron density distribution. The calculations were performed using Gaussian 16 rev. C01 [69].

For the bulk solid, the calculations of scheme BLYP (functional of Becke B88 [70] combined with the Lee–Yang–Parr correlation functional LYP [71]), different gradient-corrected functionals PBE [72] (Perdew, Burke, Ernzerhof), RPBE [73] (revised Perdew, Burke, Ernzerhof), WC [74] (Wu and Cohen), PBEsol [75] (Perdew et al.), and PW91 (Perdew et al.) [76,77] with numerical radial functions basis—double-zeta without and plane-wave basis sets with an energy cutoff of 500 eV, were probed. The GGA (generalised gradient approximation) functionals (often called nonlocal) depend on both *dρ/dr* and *ρ*, which provides an increase in accuracy but with an additional cost. The plane-wave basis guarantees a monotonic convergence to the target wavefunction and does not exhibit a basis-set superposition error. A reciprocal space-based technique, useful in the evaluation of long-range interactions, was applied. The sampling of the Brillouin zone was carried out with the scheme of Monkhorst and Pack [78] (1 × 1 × 1 or 3 × 3 × 3 k-point separations) code. Because the components of the electric field gradient (EFG) tensor are highly sensitive to the structure quality, including well-determined positions of hydrogen atoms [28,29], the FPV structure was optimized upon the assumption of a fixed unit cell. All solid-state quantum chemical calculations were carried out within the CASTEP [79] code.

In both above-described approaches, the components of the electric field gradient (EFG) tensor were calculated. The principal components of a second-rank symmetrical electric field gradient (EFG) tensor, $q_{ii}=\frac{\partial^{2}V\left(r\right)}{\partial x_{i}^{2}}$ (*i* = x, y and z; *V*(*r*)—external electrostatic potential, satisfying the relationship $\left|q_{xx}\right|\leq \left|q_{yy}\right|\leq \left|q_{zz}\right|$), were obtained after the diagonalization of the following tensor, calculated at the selected level of theory:

(1) $$\nabla E_{ij}=-\frac{1}{4\pi \varepsilon_{0}}\overset{\infty }{\underset{-\infty }{\int }}\left[\frac{3r_{i}r_{j}-\delta_{ij}{\left|r\right|}^{2}}{{\left|r\right|}^{5}}\right]\rho \left(r\right)dr$$

where (*r*) is the electron density, *r_i_* is the projection of the *r* vector onto x,y, and z-axes, and *δ_ij_* is Dirac’s delta function. Although the EFG tensor consists of six independent components in the principal axis system, it is fully described by the three principal components and the three eigenvectors describing the orientation of the principal axes with respect to an arbitrary frame. Because it is traceless, only two of the eigenvalues are mutually independent. Therefore, EFG can be described with only two parameters: the quadrupole coupling constant *e^2^qQ/h* and the asymmetry parameter *η*. Both are related to the ^14^N NQR frequencies, which are observable, according to the following equations:

(2) $$\left|\frac{e^{2}Qq}{h}\right|=\frac{2}{3}\left(\nu_{+}+\nu_{-}\right)$$

(3) $$\eta =\frac{3\left(\nu_{+}-\nu_{-}\right)}{\nu_{+}+\nu_{-}}$$

The ^14^N NQR resonance frequencies can be derived from these parameters using the set of equations:

(4) $$\nu_{+}=\left|\frac{e^{2}Qq}{4h}\right|\left(3+\eta \right)$$

(5) $$\nu_{-}=\left|\frac{e^{2}Qq}{4h}\right|\left(3-\eta \right)$$

(6) $$\nu_{0}=\nu_{+}-\nu_{-}=\left|\frac{e^{2}Qq}{2h}\right|\eta$$

A nuclear quadrupole moment for ^14^N equal to 2.044 fm^2^ [80] was assumed.

### 4.3. Quantum Theory of Atoms in Molecules (QTAIM)

A theoretical analysis of the topology of intermolecular interactions was performed within Bader’s quantum theory of atoms in molecules (QTAIM) [31] approach. The DFT wavefunction was calculated using the M062X functional and all-electron 6–311 + G(d,p) basis set. The analysis of the stationary points (maxima, saddle points, or minima in the electron density) permits differentiation of the nucleus-, bond-, ring-, and cage critical points, denoted as NAP (Nuclear Critical Point), BCP (Bond Critical Point), RCP (Ring Critical Point), and CCP (Cage Critical Point). The so-called topological descriptors calculated at the BCPs include the electron density at BCP, *ρ*(r_BCP_), three eigenvalues of the principal components of a Hessian matrix composed of second partial derivatives of the electron density describing the curvature of the electron density according to the principal axes λ_1_, λ_2_ and λ_3_, Laplacian (the sum of Hessian eigenvalues), Δ*ρ*, electron density at the bond critical point, BCP, (*ρ*_BCP_(r)), its Laplacian Δ*ρ*_BCP_(r), the potential electron energy density, *V*_BCP_(r), the kinetic electron energy density, *G*_BCP_(r), and the total electron energy density, *H*_BCP_(r). Some of these descriptors inform about the nature of the interaction (λ_2_ and Δ*ρ*), while the others (λ_3_, *V*_BCP_(r), *G*_BCP_(r), and *H*_BCP_(r)) allow a bond strength estimation. The analysis of the interactions according to QTAIM must be performed attentively because the results can be easily overinterpreted, especially in terms of the number or the nature of bonds. Therefore, the set of quantum indicators characterizing the bonds or sets of rules must be carefully analysed, especially in the case of intramolecular hydrogen bonds [81]. Despite the valid criticisms, experimental binding strengths are often in line with the model’s predictions. Thus, the hydrogen bond energy can be estimated using the well-known formulas derived from using the partitioning of the electron density scheme [82]

(7) $$E_{HB}\approx E_{E}=0.5V\left(r_{BCP}\right)$$

by Espinosa−Molins−Lecomte [83],

(8) $$E_{HB}\approx E_{M}=-0.429G\left(r_{BCP}\right)$$

by Matta [84],

(9) $$E_{HB}\approx E_{EM}=-223.08\rho \left(r_{BCP}\right)+0.7423$$

by Emamian (for neutral complexes) [85],

(10) $$E_{HB}\approx E_{A}=-\left(0.277\left|V\left(r_{BCP}\right)\right|+0.45\right)=-(0.554|E_{HBE}|+0.45)$$

by Afonin [86],

(11) $$E_{HB\;OHO}\approx E_{G}=-43.8+0.38\theta_{OHO}\exp(-5.1\left(R_{OHO}-2.49\right)$$

by Gili (for O-H···O) [52], and

(12) $$E_{N}=\{\begin{array}{c} -3.09+239\rho \left(r_{BCP}\right)\;for\;OH\cdot \cdot \cdot O\\ -2.03+225\rho \left(r_{BCP}\right)\;for\;NH\cdot \cdot \cdot O \end{array}$$

by Nikolaienko [87].

In many cases, the NCI (noncovalent interaction) based on the reduced electron density gradient (*RDG*) helps to detect interactions which were uncertain or not revealed by QTAIM [32].

The reduced electron density gradient is defined as follows:

(13) $$RDG\left(r\right)=\frac{\left|\nabla \rho (r)\right|}{2{\left(3\pi \right)}^{1/3}{\left(r\right)}^{4/3}}$$

where ∇*ρ*(*r*) is the electron density gradient and *ρ*(*r*) is electron density. The *RDG*(*r*) vs. sign(λ_2_)*ρ*(*r*) plot reveals characteristic spikes in the low-gradient and low-density regions as long as the noncovalent contacts are present in the crystalline structure. The nature of these contacts can be determined using the sign of λ_2_, which allows their classification as attractive (stabilizing; λ_2_ < 0) or repulsive (destabilizing; λ_2_ > 0). A spike in the low-gradient, low-density region at λ_2_ < 0 suggests a stabilizing interaction, such as a hydrogen bonding; a smaller spike accompanied by only a slightly negative λ_2_ indicates a weakly stabilizing interaction; and a spike associated with λ_2_ > 0 indicates the absence of noncovalent interactions. In many cases, spikes do not reach s(r) = 0, indicating that the interactions were missed by QTAIM due to a failure to detect critical points.

### 4.4. Hirshfeld Surfaces (3D HS)

The exploration of intermolecular interaction patterns and packing capacities in the solid was performed within the 3D Hirshfeld surfaces (3D HS) approach [34]. Three-dimensional HS is defined as the outer contour of the space that a molecule occupies in a crystalline environment. It is constructed with the use of the molecular weight function (a quotient of the promolecule and procrystal electron density). The descriptors such as d_norm_, shape index and curvedness of the surface mapped over 3D HS were evaluated [33]. The intermolecular interactions were visualized in the d_norm_ surface mapped over 3D HS in a red–white–blue scheme, where red was used for short contacts such as hydrogen bonds, white for contacts of about van der Waals radii, and blue for the remaining, much longer, contacts. The shape index and curvedness of the surface mapped over 3D HS described its flatness. The decomposition of the 3D HS into a 2D ‘molecular fingerprint’ (2D FP) map (plot of the distances of each surface point to the nearby interior and exterior atoms *d*_i_ versus *d*_e_) [34] which summarizes the distribution of interactions of the molecule with its environment, was made. The surface contact data derived from the 2D FP were used to obtain the enrichment ratio, E_XY_, a descriptor defined as the ratio of the proportion of the actual contacts in a crystal and the theoretical proportion of random contacts. It describes the propensity to form or avoid contacts [88].

A further global quantitative characterization of the noncovalent interactions was delivered by the electrostatic potential (ESP) surface V_S_(r) (the molecular surface defined by electron density ρ(r) = 0.001 electron bohr^3^) mapped onto a 3D HS. Using this technique, a complete description of interactions based on the electrostatic complementarity within the so-called Politzer [35,89,90] GIPF (generalised interactions properties function) approach was possible.

### 4.5. Molecular Docking

Molecular docking is a widely used technique in the screening of novel therapeutic agents, which requires not only the knowledge of the ligand structure but also the reliable 3D X-ray crystal structure of the protein. The conversion of the files with receptor and ligand structures to the .pdbqt format was made with MGLTools. The allosteric sites were identified using Allosite-Pro [57] and PASSer [58]. Molecular docking was performed using AutoDock [91] and AutoDock Vina98Vina [92]. Template docking and docking with defined searched space around the active site were probed. The best pose was selected based on the scoring function estimating the protein–ligand binding energy. The score, being a linear combination of steric, van der Waals, hydrogen bonding, electrostatic terms, torsion, and sp2-sp2 terms, was used. In the next step, the residues (side chains) closest to the active ligand were minimised with respect to the pose found, and the ligand was energy-minimised using standard potentials.

## 5. Conclusions

In the crystal, the –NH_2_ group participates in two intermolecular hydrogen bonds of different strengths N–H···N and N–H···O, –N(4)= participates in a N–H···N intermolecular hydrogen bond, but –N(1)= only in a very weak putative intramolecular hydrogen bond N–H···N (revealed only in NQR spectra, local 2D FP, and RDG). The –NH_2_ group plays a dominant role in the intermolecular bonds, irrespective of the environment (crystal or protein). In the crystal, it participates in intermolecular hydrogen bonds N–H···N and N–H···O, in the precatalytic state only in N–H···O, while in the active state in N–H···N and N–H···O, so the same binding motives are kept. Its involvement in intramolecular weak N–H⋯N and strong N–H⋯O hydrogen bonds seems to be an advantage when it comes to planarity and stacking (needed for the mimicking of guanosine), but it is an obstacle that weakens the bond pairing. The –N(1)= nitrogen plays only a supportive role, and -N(4)= is excluded from binding in the active form due to its substitution. This is reflected in the asymmetry parameters—the highest for asymmetrically bonded –NH_2_ and the lowest for –N(4)= participating only in one bond coaxial with the Z-axis of the EFG tensor. The ordering of the most important interactions according to the decreasing energy in the crystals is as follows:

O–H⋯O > N–H⋯O ≈ π⋯π stacking (parallelly oriented molecules) > N–H⋯N ≈ ≈ π⋯π stacking (flipped molecules) > C–H⋯O >> F⋯F > F–H⋯C,

among which N–H⋯O, N–H⋯N, π⋯π stacking, and possibly F–H⋯C, are present also upon binding with the template RNA. The fluorine in this structure plays a minor role and affects only the conformation.

Considering the cofactor binding strength, three ligands FVP-RMP and two FVP-RTP can be ordered as FVP-RTP (active state) > FVP-RTP (precatalytic state) > FVP-RMP. The total binding affinity (between target protein and ligand) is the highest for FVP-RTP (−494.30 kJ/mol; active state), followed by FVP-RTP (−471.58 kJ/mol; precatalytic state) and FVP-RMP (−247.02 kJ/mol). The total binding affinity for FVP-RTP in allosteric pocket is as high as −402.96 kJ/mol. Strong direct binding of FVP-RTP to both the active site and cofactor suggests a possible alternative, allosteric mechanism of FVP action, which may explain the scattering of the results of clinical trials or the synergistic effect observed in combined treatment against SARS-CoV-2.

## Figures and Tables

**Table 1:** The experimental ^14^N NQR parameters for favipiravir (FVP).

Site	ν_+_ [MHz]	ν_−_ [MHz]	ν_0_ [MHz]	|e^2^qQ/h|[MHz]	η	T [K]	Final Assignment *
pyrazine nitrogens	3.865	2.965	0.900	4.553	0.395	295	−N(1)=
	3.570	2.950	0.620	4.347	0.285		−N(4)=
amide nitrogen	2.060	1.560	0.500	2.413	0.414		−NH_2_
pyrazine [44]	4.329	3.017	1.312	4.897	0.536	295	−N=

* The final assignment made was based on quantum chemical calculations.

**Table 2:** The ^14^N NQR parameters calculated at the GGA/RPBE level of theory assuming the single molecule (monomer) of FVP, “parental” pyrazine and its derivatives (geometry optimized at the same level).

Substituent	Site	ν_+_ [MHz]	ν_−_ [MHz]	ν_0_ [MHz]	e^2^qQ/h [MHz]	η	Correlation
FPV	−N(1)=	3.744	3.043	0.701	−4.538	0.300	ν_GGA_ = 1.025ν_NQR_ + 0.203 r^2^ = 0.8685; s = 1.8060
	−N(4)=	3.902	3.214	0.688	−4.731	0.290	
	−NH_2_	2.997	2.676	0.321	−3.789	0.160	
pyrazine	−N=	4.646	3.097	1.549	−5.162	0.600	ν_GGA_ = 1.021ν_NQR_ + 0.152 r^2^ = 0.9887; s = 0.0271
2-fluoro-pyrazine	−N(1)=	4.403	3.460	0.944	−5.242↑	0.36↓	experimental data unavailable
	−N(4)=	4.082	2.916	1.166	−4.665↓	0.50↓	
2-hydroxy-pyrazine	−N(1)=	4.279	3.548	0.731	−5.218↑	0.28↓	
	−N(4)=	4.049	2.872	1.177	−4.614↓	0.51↓	
pyrazine-2-carboxamide	−N(1)=	3.826	2.771	1.056	−4.398↓	0.48↓	
	−N(4)=	3.603	2.869	0.734	−4.315↓	0.34↓	
	−NH_2_	3.087	2.831	0.256	−3.945↓	0.13↓	

↑↓—increase and decrease relative to the unsubstituted pyrazine.

**Table 3:** The ^14^N NQR parameters calculated theoretically assuming single molecule and crystal (polymorph I) of FVP.

Structure, CCDC	Site	ν_+_ [MHz]	ν_−_ [MHz]	ν_0_ [MHz]	e^2^qQ/h [MHz]	η	Calculations Level	Correlation
2106318, 100 K monomer	−N(1)=	3.772	2.964	0.808	−4.491	0.36	GGA/RPBE	ν_GGA_ = 1.005ν_NQR_ + 0.188 r^2^ = 0.9647; s = 0.4256
	−N(4)=	4.055	3.186	0.869	−4.827	0.36		
	−NH_2_	2.558	1.943	0.615	−3.001	0.41		
2106318, 100 K solid	−N(1)=	3.885	2.971	0.914	−4.570	0.40	GGA/PBEsol	ν_GGA_ = 0.989ν_NQR_ + 0.042 r^2^ = 0.9686; s = 0.3650
	−N(4)=	3.759	2.994	0.765	−4.502	0.34		
	−NH_2_	1.938	1.119	0.819	−2.038	0.80		
	−N(1)=	3.938	3.052	0.885	−4.660	0.38	GGA/PBE	ν_GGA_ = 1.009ν_NQR_ + 0.054 r^2^ = 0.9756; s = 0.2937
	−N(4)=	3.809	3.075	0.734	−4.589	0.32		
	−NH_2_	2.075	1.219	0.856	−2.196	0.78		
	−N(1)=	3.958	3.047	0.911	−4.670	0.39	GGA/WC Wu-Cohen	ν_GGA_ = 1.007ν_NQR_ + 0.057 r^2^ = 0.9686; s = 0.3892
	−N(4)=	3.835	3.054	0.781	−4.593	0.34		
	−NH_2_	2.021	1.153	0.868	−2.116	0.82		
	−N(1)=	4.008	3.086	0.922	−4.729	0.39	GGA/PW91	ν_GGA_ = 1.023ν_NQR_ + 0.053 r^2^ = 0.9754; s = 0.3041
	−N(4)=	3.870	3.103	0.767	−4.649	0.33		
	−NH_2_	2.071	1.225	0.846	−2.197	0.77		
	−N(1)=	3.895	3.060	0.835	−4.637	0.36	GGA/RPBE	ν_GGA_ = 1.009ν_NQR_ + 0.047 r^2^ = 0.9827; s = 0.2068
	−N(4)=	3.762	3.078	0.684	−4.560	0.30		
	−NH_2_	2.133	1.308	0.826	−2.294	0.720		
X-ray 2048895 100 K monomer	−N(1)=	3.796	2.942	0.853	−4.492	0.38	GGA/RPBE	ν_GGA_ = 1.001ν_NQR_ + 0.203 r^2^ = 0.9654; s = 0.4143
	−N(4)=	4.086	3.168	0.919	−4.836	0.38		
	−NH_2_	2.539	1.942	0.597	−2.987	0.40		
X-ray 2048895 100 K solid	−N(1)=	3.896	2.959	0.937	−4.570	0.41	GGA/PBESol	ν_GGA_ = 0.984ν_NQR_ + 0.061 r^2^ = 0.9643; s = 0.4133
	−N(4)=	3.792	2.979	0.813	−4.514	0.36		
	−NH_2_	1.933	1.103	0.830	−2.024	0.82		
	−N(1)=	3.949	3.041	0.909	−4.660	0.39	GGA/PBE	ν_GGA_ = 1.008ν_NQR_ + 0.061 r^2^ = 0.9756; s = 0.2933
	−N(4)=	3.842	3.060	0.782	−4.601	0.34		
	−NH_2_	2.051	1.222	0.829	−2.182	0.76		
	−N(1)=	3.980	3.023	0.957	−4.669	0.41	GGA/WC Wu-Cohen	ν_GGA_ = 1.005ν_NQR_ + 0.068 r^2^ = 0.9677; s = 0.3888
	−N(4)=	3.868	3,039	0.829	−4.605	0.36		
	−NH_2_	1.996	1.156	0.840	−2.101	0.80		
	−N(1)=	4.021	3.075	0.946	−4.731	0.40	GGA/PW91	ν_GGA_ = 1.021ν_NQR_ + 0.061 r^2^ = 0.9840; s = 0.3222
	−N(4)=	3.904	3.088	0.816	−4.661	0.35		
	−NH_2_	2.049	1.221	0.828	−2.180	0.76		
	−N(1)=	3.908	3.049	0.858	−4.638	0.37	GGA/RPBE	ν_GGA_ = 1.009ν_NQR_ + 0.052 r^2^ = 0.9836; s = 0.1955
	−N(4)=	3.796	3.065	0.732	−4.574	0.32		
	−NH_2_	2.108	1.310	0.798	−2.279	0.70		
X-ray 969968 296 K monomer	−N(1)=	3.852	2.966	0.886	−4.545	0.39	GGA/RPBE	ν_GGA_ = 0.994ν_NQR_ + 0.239 r^2^ = 0.9696; s = 0.3569
	−N(4)=	4.113	3.145	0.968	−4.839	0.40		
	−NH_2_	2.553	1.849	0.704	−2.935	0.48		
X-ray 969968 296 K solid	−N(1)=	3.941	2.973	0.968	−4.609	0.42	GGA/PBESol	ν_GGA_ = 0.980ν_NQR_ + 0.108 r^2^ = 0.9572; s = 0.4941
	−N(4)=	3.849	2.984	0.865	−4.555	0.38		
	−NH_2_	1.999	1.092	0.907	−2.061	0.88		
	−N(1)=	3.988	3.050	0.938	−4.692	0.40	GGA/PBE	ν_GGA_ = 0.999ν_NQR_ + 0.110 r^2^ = 0.9671; s = 0.3911
	−N(4)=	3.880	3.049	0.831	−4.619	0.36		
	−NH_2_	2.110	1.187	0.923	−2.198	0.84		
	−N(1)=	4.016	3.050	0.966	−4.711	0.41	GGA/WC Wu-Cohen	ν_GGA_ = 1.001ν_NQR_ + 0.1173 r^2^ = 0.9630; s = 0.4437
	−N(4)=	3.918	3.057	0.860	−4.650	0.37		
	−NH_2_	2.099	1.164	0.935	−2.175	0.86		
	−N(1)=	4.067	3.089	0.978	−4.771	0.41	GGA/PW91	ν_GGA_ = 1.017ν_NQR_ + 0.108 r^2^ = 0.9673; s = 0.4026
	−N(4)=	3.962	3.092	0.870	−4.703	0.37		
	−NH_2_	2.113	1.206	0.907	−2.213	0.82		
	−N(1)=	3.951	3.063	0.888	−4.676	0.38	GGA/RPBE	ν_GGA_ = 1.004ν_NQR_ + 0.098 r^2^ = 0.9779; s = 0.2628
	−N(4)=	3.854	3.069	0.785	−4.615	0.34		
	−NH_2_	2.170	1.292	0.877	−2.308	0.76		

**Table 4:** The ^14^N NQR parameters calculated theoretically assuming single molecule and crystal (polymorph II) of FVP.

Structure, CCDC	Site	ν_+_ [MHz]	ν_−_ [MHz]	ν_0_ [MHz]	e^2^qQ/h [MHz]	η	Calculations Level	Correlation
X-ray 2047143 monomer	−N(1)=	3.800	3.046	0.753	−4.564	0.33	GGA/RPBE	ν_GGA_ = 1.014ν_NQR_ + 0.1734 r^2^ = 0.9676; s = 0.3975
	−N(4)=	4.008	3.235	0.773	−4.829	0.32		
	−NH_2_	2.604	1.848	0.757	−2.968	0.51		
X-ray 2047143 solid	−N(1)=	3.965	3.052	0.912	−4.678	0.42	GGA/PBESol	ν_GGA_ = 0.998ν_NQR_ + 0.060 r^2^ = 0.9589; s = 0.4912
	−N(4)=	3.714	3.059	0.655	−4.555	0.38		
	−NH_2_	2.063	1.078	0.984	−2.061	0.88		
	−N(1)=	4.017	3.135	0.882	−4.768	0.37	GGA/PBE	ν_GGA_ = 1.023ν_NQR_ + 0.050 r^2^ = 0.9667; s = 0.4165
	−N(4)=	3.744	3.147	0.597	−4.594	0.26		
	−NH_2_	2.177	1.189	0.987	−2.244	0.88		
	−N(1)=	4.038	3.130	0.908	−4.779	0.38	GGA/WC Wu-Cohen	ν_GGA_ = 1.019ν_NQR_ + 0.062 r^2^ = 0.9616; s = 0.4768
	−N(4)=	3.788	3.121	0.668	−4.606	0.29		
	−NH_2_	2.129	1.129	0.999	−2.172	0.92		
	−N(1)=	4.092	3.172	0.920	−4.843	0.38	GGA/PW91	ν_GGA_ = 1.037ν_NQR_ + 0.052 r^2^ = 0.9669; s = 0.4233
	−N(4)=	3.805	3.177	0.628	−4.655	0.27		
	−NH_2_	2.179	1.190	0.988	−2.246	0.88		
	−N(1)=	3.969	3.140	0.829	−4.739	0.35	GGA/RPBE	ν_GGA_ = 1.023ν_NQR_ + 0.039 r^2^ = 0.9730; s = 0.3354
	−N(4)=	3.694	3.146	0.547	−4.560	0.24		
	−NH_2_	2.225	1.279	0.946	−2.336	0.81		

**Table 5:** The ^14^N NQR parameters calculated theoretically assuming optimized geometry of FVP.

Structure, CCDC	Site	ν_+_ [MHz]	ν_−_ [MHz]	ν_0_ [MHz]	e^2^qQ/h [MHz]	η	Calculations Level	Correlation
X-ray 2048895 optimized at RPBE with fixed cell parameters	−N(1)=	3.898	3.062	0.835	−4.640	0.360	GGA/PBESol	ν_GGA_ = 1.013ν_NQR_ − 0.484 r^2^ = 0.9960; s = 0.0478
	−N(4)=	3.507	2.948	0,559	−4.303	0.260		
	−NH_2_	1.995	1.415	0.580	−2.273	0.510		
	−N(1)= −N(4)=	3.954	3.149	0.805	−4.735	0.34	GGA/PBE	ν_GGA_ = 1.040ν_NQR_ − 0.0545 r^2^ = 0.9964; s = 0.0526
		3.547	3.042	0.505	−4.393	0.23		
	−NH_2_	2.129	1.563	0.566	−2.461	0.46		
	−N(1)=	3.983	3.130	0.854	−4.742	0.360	GGA/WC Wu-Cohen	ν_GGA_ = 1.035ν_NQR_ − 0.046 r^2^ = 0.9967; s = 0.0461
	−N(4)=	3.564	3.016	0.548	−4.387	0.250		
	−NH_2_	2.067	1.476	0.591	−2.362	0.500		
	−N(1)=	4.026	3.185	0.841	−4.807	0.350	GGA/PW91	ν_GGA_ = 1.053ν_NQR_ − 0.053 r^2^ = 0.9972; s = 0.0767
	−N(4)=	3.604	3.070	0.534	−4.449	0.240		
	−NH_2_	2.132	1.565	0.567	−2.465	0.460		
	−N(1)=	3.911	3.157	0.754	−4.712	0.320	GGA/RPBE	ν_GGA_ = 1.041ν_NQR_ − 0.055 r^2^ = 0.9919; s = 0.1022
	−N(4)=	3.512	3.052	0.459	−4.376	0.210		
	−NH_2_	2.200	1.660	0.540	−2.573	0.420		
2106318 100 K optimized at RPBE with fixed cell parameters	−N(1)=	3.892	3.058	0.834	−4.633	0.360	GGA/PBESol	ν_GGA_ = 1.011ν_NQR_ − 0.042 r^2^ = 0.9967; s = 0.0388
	−N(4)=	3.511	2.951	0.560	−4.308	0.260		
	−NH_2_	2.015	1.439	0.576	−2.303	0.500		
	−N(1)=	3.948	3.144	0.804	−4.728	0.340	GGA/PBE	ν_GGA_ = 1.039ν_NQR_ − 0.049 r^2^ = 0.9959; s = 0.0518
	−N(4)=	3.552	3.046	0.506	−4.399	0.230		
	−NH_2_	2.148	1.588	0.560	−2.491	0.450		
	−N(1)=	3.977	3.125	0.852	−4.735	0.360	GGA/WC Wu-Cohen	ν_GGA_ = 1.033ν_NQR_ − 0.038 r^2^ = 0.9975; s = 0.0314
	−N(4)=	3.580	3.009	0.571	−4.393	0.260		
	−NH_2_	2.082	1.508	0.574	−2.393	0.480		
	−N(1)=	4.017	3.178	0.839	−4.797	0.350	GGA/PW91	ν_GGA_ = 1.054ν_NQR_ − 0.051 r^2^ = 0.9975; s = 0.0714
	−N(4)=	3.609	3.075	0.535	−4.456	0.240		
	−NH_2_	2.146	1.597	0.549	−2.495	0.440		
	−N(1)=	3.904	3.152	0.753	−4.704	0.320	GGA/RPBE	ν_GGA_ = 1.040ν_NQR_ − 0.051 r^2^ = 0.9924; s = 0.1252
	−N(4)=	3.509	3.050	0.459	−4.373	0.210		
	−NH_2_	2.212	1.691	0.520	−2.602	0.400		

**Table 6:** The ^14^N NQR parameters calculated at M062X/6-311 + G** level for a single molecule and cluster of FVP.

Structure, CCDC	Site	ν_+_ [MHz]	ν_−_ [MHz]	ν_0_ [MHz]	e^2^qQ/h [MHz]	η	Correlation
2048895 monomer	−N(1)=	4.251	3.170	1.081	−4.947	0.437	ν_DFT_ = 1.116ν_NQR_ + 0.241 r^2^ = 0.9197; s = 1.2453
	−N(4)=	4.503	3.469	1.034	−5.315	0.389	
	−NH_2_	2.930	2.659	0.270	−3.726	0.145	
2048895 cluster	−N(1)=	4.475	3.280	1.194	−5.170	0.462	ν_DFT_ = 1.125ν_NQR_ + 0.040 r^2^ = 0.9926; s = 0.1115
	−N(4)=	4.235	3.348	0.887	−5.055	0.351	
	−NH_2_	2.247	1.752	0.495	−2.666	0.371	

**Table 7:** Percentage contributions to the 3D Hirshfeld surface area calculated for each pair of species.

Structure, CCDC	Homonuclear					Heteronuclear *									
	C···C	F···F	N···N	O···O	H···H	C···F	C···H	C···N	C···O	F···H	F···N	F···O	N···H	O···H	N···O
2106318 X-ray	0	3.4	3.8	0.1	12.0	1.3	10.1	8.2	7.1	15.6	0	4.2	11.5	17.8	4.8
2106318 optimized protons	0	3.4	3.8	0.1	12.0	1.4	10.0	8.1	7.1	15.6	0	4.2	11.5	17.8	4.8
2106318 full optimization	0.3	4.1	3.9	0.3	12.1	1.4	9.2	8.2	7.7	14.9	0	4.3	11.7	17.0	5.0
2048895 X-ray	0	3.4	3.8	0.1	12	1.2	10	8.4	7.1	15.6	0	4.2	11.5	17.8	4.8
2048895 optimized protons	0	3.4	3.8	0.1	12	1.4	9.8	8.3	7.1	15.6	0	4.2	11.5	17.8	4.8
2048895 full optimization	0	3.4	3.8	0.1	12	1.4	9.6	8.3	7.1	15.6	0	4.2	11.5	17.8	4.8
969968 X-ray	0	3.4	3.8	0.1	12	1.2	9.7	8.2	7.1	15.6	0	4.2	11.5	17.8	4.8
969968 optimized protons	0	3.4	3.8	0.1	12	1.2	10	8.4	7.1	15.6	0	4.2	11.5	17.8	4.8

* The contributions from X···Y and Y···X, where X, Y = C, F, N, O, and H are summarized.

**Table 8:** Enrichment ratios E_XY_ characterizing the various contacts in FVP.

Structure, CCDC	Atom	C	F	H	N	O
2106318 X-ray	Surface %	13.35	13.95	39.5	16.5	17.05
	C	0	-	-	-	-
	F	0.35	1.75	-	-	-
	H	0.96	1.42	0.77	-	-
	N	1.91	0.00	0.91	1.48	-
	O	1.56	0.88	1.32	0.88	0.03
2106318 optimized protons	Surface %	13.3	14.0	39.5	16.0	17.5
	C	0	-	-	-	-
	F	0.38	1.73	-	-	-
	H	0.95	1.41	0.77	-	-
	N	1.90	0.00	0.91	1.48	-
	O	1.57	0.88	1.32	0.88	0.03
2106318 full optimization unit cell fixed	Surface %	13.55	14.4	38.5	16.3	17.3
	C	0.16	-	-	-	
	F	0.36	1.98	-	-	
	H	0.88	1.34	0.82	-	
	N	1.85	0.00	0.93	1.46	
	O	1.64	0.86	1.28	0.88	0.10
2048895 X-ray	Surface %	13.35	13.9	39.45	16.15	17.05
	C	0	-	-	-	-
	F	0.32	1.76	-	-	-
	H	0.95	1.42	0.77	-	-
	N	1.95	0	0.90	1.46	-
	O	1.56	0.89	1.32	0.87	0.03
2048895 optimized protons	Surface %	13.3	14.0	39.35	16.1	17.05
	C	0	-	-	-	-
	F	0.38	1.73	-	-	-
	H	0.94	1.42	0.77	-	-
	N	1.94	0	0.91	1.47	-
	O	1.57	0.88	1.33	0.87	0.03
2048895 full optimization unit cell fixed	Surface %	13.2	14.0	39.25	16.1	17.05
	C	0	-	-	-	-
	F	0.38	1.73	-	-	-
	H	0.93	1.42	0.78	-	-
	N	1.95	0	0.91	1.47	-
	O	1.58	0.88	1.33	0.87	0.03
969968 X-ray	Surface %	13.1	13.9	39.3	16.05	17.05
	C	0	-	-	-	-
	F	0.33	1.76	-	-	-
	H	0.94	1.43	0.78	-	-
	N	1.95	0	0.91	1.48	-
	O	1.59	0.89	1.33	0.88	0.03
969968 optimized protons	Surface %	13.35	13.9	39.45	16.15	17.05
	C	0	-	-	-	-
	F	0.32	1.76	-	-	-
	H	0.95	1.42	0.77	-	-
	N	1.95	0	0.90	1.46	-
	O	1.56	0.89	1.32	0.87	0.03

**Table 9:** Percentage contributions to the 3D Hirshfeld surface area calculated for single species.

Contact Type	–N(1)=	–N(4)=	–NH_2_	Contact Type	=O	–OH	Contact Type	F
N···C	59.9	59.7	19.3	O···C	22.9	30	F···C	29.3
N···H	20.4	35.2	80.7	O···H	76.4	51.5	F···H	43.1
N···O	9.2	1	-	O···O	0	0	F···O	8.2
N···N	5.3	4.1	-	O···N	0	9.7	F···N	1.6
N···F	5.1	-	-	O···F	0.7	8.9	F···F	17.9

**Table 10:** The total interaction energy partitions the interactions into electrostatic, **E_el_.*,*** polarization (induction), **E_p_,** dispersion, **E_d_**, and repulsion (exchange), **E_r_**, terms.

Interaction	E_el_. [kJ/mol]	E_p_ [kJ/mol]	E_d_ [kJ/mol]	E_r_ [kJ/mol]	E_total_ [kJ/mol]	Structure, CCDC
N–H⋯O	−37.6	−5.9	−6.9	25.6	−24.8	2048895 optimized
N–H⋯N	−22.0	−5.6	−10.8	18.7	−19.6	
C–H⋯O	−6.0	−1.1	−5.0	4.5	−7.6	
F⋯F	−1.2	−0.1	−4.4	1.2	−4.6	
π⋯π stacking (flip)	−2.5	−1.2	−20.8	7.5	−17.0	
π⋯π stacking (parallel)	−4.1	−1.3	−21.1	7.5	−19.0	
N–H⋯O	−25.7	−3.9	−6.4	13.1	−22.9	2048895 X-ray
N–H⋯N	−4.9	−1.1	−21.2	6.8	−20.4	
C–H⋯O	−4.0	−0.9	−4.5	2.2	−7.4	
F⋯F	−1.4	−0.1	−3.0	0.1	−4.5	
π⋯π stacking (flip)	−14.0	−3.9	−10.7	11.7	−16.8	
π⋯π stacking (parallel)	−3.2	−1.0	−21.2	7.4	−17.9	

**Table 11:** Topological parameters describing the interactions in FVP (electron density at the bond critical point, BCP, and (ρ_BCP_(r)), its Laplacian (Δρ_BCP_(r)), the potential electron energy density (V_BCP_(r)), the kinetic electron energy density (G_BCP_(r)), the total electron energy density (H_BCP_(r)), the bonding energy according to Espinosa (E_E_), Matta (E_M_), Emamian (E_EM_), Afonin (E_A_), Gilli (E_G_) and Nikolaienko (E_N_) calculated at the M062X/6-311 + G** level.

Interaction		Nitrogen Site	R_X_⋯_Y_, <XHY	ρ_BCP_(r) [a.u.]	Δρ_BCP_(r) [a.u.]	V_BCP_ [a.u.]	G_BCP_ [a.u.]	E_E_ [kJ/mol]	E_M_ [kJ/mol]	E_EM_ [kJ/mol]	E_A_ [kJ/mol]	E_G_ [kJ/mol]	E_N_ [kJ/mol]
hydrogen bonds	N-H⋯N *	-NH_2_, -N(1)=	2.321, 101.8	-	-	-	-	-	-	-	-	-	-
	O-H⋯O	-	2.591, 142.6	0.042481	0.143112	−0.04196	0.038869	−55.08	−43.77	−36.54	−30.97	−25.92	−29.55
hydrogen bonds	N–H⋯O	-NH_2_	2.880, 166.9	0.026747	0.110269	−0.02233	0.024948	−29.31	−28.10	−21.86	−16.69	-	−16.69
	N–H⋯N	-NH_2_, -N(4)=	2.961, 133.8	0.018429	0.06814	−0.01189	0.014461	−15.61	−16.29	−14.10	-	-	-
	C–H⋯O	-	3.420, 148.9	0.009161	0.030523	−0.00555	0.006589	−7.28	−7.42	−5.44	−4.48	-	-
	F–H⋯C	-	3.459, 118.8	0.00343	0.015936	−0.00238	0.003182	−3.12	−3.58	−0.10	−2.18	-	-
	F⋯F	-	3.067	0.003874	0.021766	−0.00353	0.004488	−4.64	−5.06	-	-	-	-
π⋯π stacking (flip)	F⋯C	-	6.535	0.002768	0.012361	−0.00158	0.002333	−2.06	-	-	-	-	-
	N⋯N	-N(1)=, -N(4)=	6.166	0.005919	0.018211	−0.00326	0.003905	−4.28	-	-	-	-	-
	N⋯O	-NH_2_	6.472	0.005106	0.016997	−0.00309	0.003668	−4.05	-	-	-	-	-
	N⋯O	-NH_2_	6.679	0.003988	0.012019	−0.00228	0.002642	−2.99	-	-	-	-	-
π⋯π stacking (parallel)	O⋯F	-	6.682	0.001989	0.010403	−0.00126	0.00193	−1.65	-	-	-	-	-
	N⋯C	-N(4)=	6.785	0.005659	0.01542	−0.00285	0.003354	−3.75	-	-	-	-	-
	O⋯C	-	6.075	0.006435	0.021031	−0.00384	0.004551	−5.04	-	-	-	-	-
	N⋯C	-N(4)=	6.404	0.003977	0.011922	−0.00208	0.002532	−2.74	-	-	-	-	-
stabilising (in a layer)	N⋯O	-N(1)=	6.385	0.004228	0.015145	−0.00252	0.003155	−3.31	-	-	-	-	-
	F⋯O	-	6.167	0.003083	0.016013	−0.00236	0.003182	−3.09	-	-	-	-	-

* not detected by QTAIM.

## Data Availability

Not applicable.
